# LLM agents overcome the machine penalty when acting fairly but not when acting selfishly or altruistically

**DOI:** 10.1093/nsr/nwag223

**Published:** 2026-04-16

**Authors:** Zhen Wang, Ruiqi Song, Chen Shen, Shiya Yin, Zhao Song, Balaraju Battu, Lei Shi, Danyang Jia, Talal Rahwan, Shuyue Hu

**Affiliations:** School of Cybersecurity, and School of Artificial Intelligence, OPtics and ElectroNics (iOPEN), Northwestern Polytechnical University, Xi’an 710072, China; School of Cybersecurity, and School of Artificial Intelligence, OPtics and ElectroNics (iOPEN), Northwestern Polytechnical University, Xi’an 710072, China; Faculty of Engineering Sciences, Kyushu University, Fukuoka 819-0395, Japan; School of Cybersecurity, and School of Artificial Intelligence, OPtics and ElectroNics (iOPEN), Northwestern Polytechnical University, Xi’an 710072, China; School of Computing, Engineering and Digital Technologies, Teesside University, Middlebrough TS1 3BX, UK; Computer Science, Science Division, New York University Abu Dhabi, Abu Dhabi 129188, UAE; School of Statistics and Mathematics, Yunnan University of Finance and Economics, Yunnan 650221, China; School of Cybersecurity, and School of Artificial Intelligence, OPtics and ElectroNics (iOPEN), Northwestern Polytechnical University, Xi’an 710072, China; Computer Science, Science Division, New York University Abu Dhabi, Abu Dhabi 129188, UAE; Department of AI for Science, Shanghai Artificial Intelligence Laboratory, Shanghai 200238, China

**Keywords:** artificial intelligence, large language model, human–machine interaction, game theory, behavioral economic

## Abstract

Despite rapid technological progress, effective human–machine cooperation remains a significant challenge. Humans tend to cooperate less with machines than with fellow humans, a phenomenon known as the machine penalty. Here, we show that artificial intelligence (AI) agents powered by large language models can overcome this penalty in social dilemma games with communication. In a pre-registered experiment with 1152 participants, we deploy AI agents exhibiting three distinct personas: selfish, cooperative and fair. However, only fair agents elicit human cooperation at rates comparable to human–human interactions. Analysis reveals that fair agents, similar to human participants, occasionally break pre-game cooperation promises but nonetheless effectively establish cooperation as a social norm. These results challenge the conventional wisdom of machines as altruistic assistants or rational actors. Instead, our study highlights the importance of AI agents reflecting the nuanced complexity of human social behaviors—imperfect yet driven by deeper social cognitive processes.

## Teaser

Fair but imperfect AI agents, capable of breaking promises, overcome human reluctance to cooperate with machines.

## INTRODUCTION

In today’s rapidly advancing technological landscape, cooperation between humans and machines is emerging as a cornerstone of societal progress and innovation. For example, Pactum’s artificial intelligence (AI) agents now negotiate on behalf of Walmart with human vendors, closing deals in just days rather than the traditional weeks or months [1]. AI coding assistants, such as GitHub Copilot, support code autocompletion and fixes while keeping humans in the loop, increasing the productivity of engineers at JPMorgan by up to 20% [2]. Machines, no longer passive tools merely executing our commands, have evolved into sophisticated, autonomous agents ready to actively work—and, more importantly, to cooperate—with humans [3,4]. This paradigm shift makes understanding and fostering human–machine cooperation imperative. Not only does it allow us to harness the complementary strengths of both [5], but it also helps navigate social dilemmas—tensions between individual and collective interests—a crucial and nearly unavoidable challenge in human–machine interactions [6].

Despite immense potential, studies have consistently identified a significant gap between human–human and human–machine cooperation, a phenomenon known as the machine penalty [7]. While this phenomenon has been widely documented in incentivized economic games [8,9], it also manifests in real-world contexts. As a common example, human drivers are more likely to deny autonomous vehicles the right of way at junctions than they are to other human drivers [10,11]. Part of this penalty stems from technical limitations: machines must be able to understand, communicate and find common ground with humans [6]. However, at its core, the machine penalty reflects a form of algorithm aversion, whereby humans are reluctant to view machines as genuine social partners [12,13]. This aversion, common across various domains, often leads to less trust in machines [14] and a tendency to assign machines greater blame for mistakes [15]. Furthermore, machines’ typical lack of cultural norms [16], moral understanding [17], emotional capacity [18] and fairness considerations [19], which are elements crucial to human cooperation, only exacerbates the aversion.

Recent efforts to mitigate the machine penalty have focused on anthropomorphism [20–22]. The rationale is that, since humans are more cooperative with fellow humans, endowing machines with human-like traits might bridge the gap. However, superficial anthropomorphic designs, such as equipping machines with emotional expressions or human-like faces [20,23], have shown little effect. Nevertheless, enhancing human-likeness can sometimes evoke feelings of uncanniness [24]. Some studies have had success with ethically questionable genderization [21,22], such as adding female cues of long hair to machines. Another approach has been to conceal machines’ true nature [8]. While this approach can effectively reduce the gap, it compromises technological transparency and can be seen as deceptive. Collectively, these findings cast doubt on whether humanization is the answer and highlight the need to rethink which elements are truly necessary for machines to foster human cooperation.

To close this gap, this paper studies human cooperation with AI agents powered by large language models (LLMs). The reasons behind this design choice are manifold. First, LLMs are well known for their ability to understand language and generate conversations, making them well suited for interactions with humans [25,26]. Second, their extensive training on human corpora allows them to grasp key concepts such as rationality, risk, trust and fairness, which underpin human cooperative behaviors [27,28]. Third, LLMs can generate diverse human simulacra by varying high-level descriptions, enabling them to emulate the nuances of human behavior [29] and enhance their cooperative potential.

We focus on prisoner’s dilemma games with pre-game communication, a stylized, controlled social dilemma in which players choose between cooperation and defection. While collective benefits depend on cooperation, individual incentives favor defection. We design agents with enhanced strategic reasoning and decision-making capabilities, and humanize them by assigning them one of three distinct personas (see the Methods section for details): (i) cooperative, aiming to assist its human associate; (ii) selfish, focusing solely on maximizing its self-interest; and (iii) fair, balancing its own and collective interest while slightly prioritizing self-interest. While selfish agents reflect the common notion of rationality [30,31] in AI and game theory, whereby agents act primarily to maximize their own payoff, cooperative agents and fair agents are designed to embody prosocial preferences [32], a prominent theoretical account of human cooperation.

Our experiment, with a total of 1152 participants, shows that fair agents are able to overcome the machine penalty, whereas cooperative and selfish agents do not. The post-experiment analysis aims to understand the mechanisms at play, focusing on differences in communication, reasoning, decision-making, the ability to evoke descriptive norms (i.e. beliefs about other participants commonly cooperating), and participants’ perceptions of agents’ minds and human-like traits. The analysis reveals that fair agents, despite occasional strategic promise breaches, successfully establish cooperative norms and foster positive human perceptions of their mind, trustworthiness and intelligence.

## RESULTS

Our experiment involves four types of treatments: human–human (H–H), human–fair agent (H–F), human–cooperative agent (H–C) and human–selfish agent (H–S) interactions. Each treatment spans 10 rounds, with participants randomly paired with a knowingly new associate in each round. Before making their choices in each round, participants and their associates exchange two rounds of messages. From the outset, we explicitly inform participants of the true nature of their associates, that is, whether they are interacting with humans or intelligent machines. We also conduct a label-uninformed setting in which the nature of the associate was not disclosed. The following results focus exclusively on the label-informed setting. Detailed experimental implementations are presented in Note S1.

### Overcoming the machine penalty

**Figure 1.**
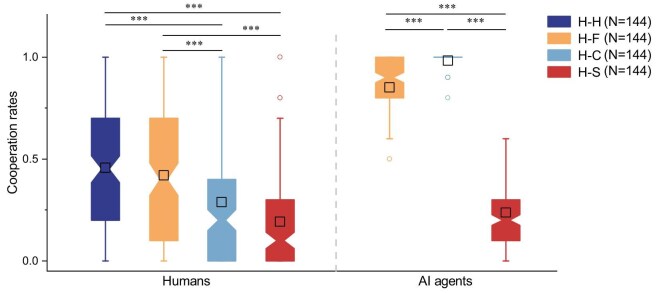
Fair agents, unlike cooperative or selfish agents, are as effective as humans at eliciting human cooperation, thereby overcoming the machine penalty. The left panel depicts participants’ cooperation rates, while the right panel depicts the cooperation rates of agents. Participants’ cooperation rates in the H–F treatment show no significant difference compared to those in the H–H treatment ($W=11\, 096$, $p=0.3$, Cohen’s $d=-0.11$). However, their cooperation rates in both the H–C and H–S treatments are significantly lower than those in the H–H treatment (H–C versus H–H: $W=7240.5$, $p<10^{-6}$, Cohen’s $d =-0.52$; H–S versus H–H: $W=5552.5$, $p<10^{-12}$, Cohen’s $d=0.97$). The cooperation rates of fair agents are significantly lower than those of cooperative agents ($W=4089$, $p<10^{-16}$, Cohen’s $d = -1.32$), but significantly higher than those of selfish agents ($W=20\, 680$, $p<10^{-16}$, Cohen’s $d = 4.29$). Two-tailed Mann–Whitney *U* tests are used for pairwise comparisons. The robustness of these results is further corroborated by a one-way ANOVA test (Table S1).

Figure 1 shows the cooperation rates of humans and agents in human–human and three types of human–agent interactions. The left panel shows that human cooperation rates in interactions with fair agents are comparable to those observed in human–human interactions. This indicates that fair agents are as effective as humans in eliciting cooperation. In contrast, human cooperation rates are significantly lower in interactions with cooperative or selfish agents than in human–human interactions. The right panel shows the cooperation rates of three types of agents. Cooperative agents consistently cooperate with minimal variability. Selfish and fair agents display within-group variability, generally showing tendencies to defect and cooperate, respectively.

Thus, AI agents powered by LLMs can, in principle, overcome the longstanding machine penalty, inducing human cooperation at levels similar to human–human interactions, even when their machine nature is fully disclosed. However, this is only true for agents with a humanized, fair persona. The cooperation gap cannot be bridged by cooperative agents, despite their purely benevolent intent, nor by selfish agents, despite their rational decision-making.

### Cooperation agreement and promise breach

**Figure 2.**
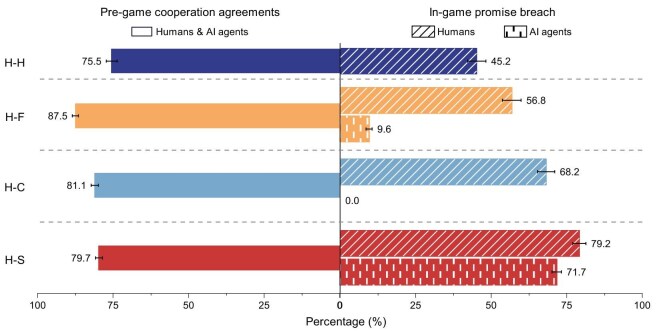
All three types of agents frequently establish cooperation agreements with humans during the pre-game communication. However, humans often break cooperation promises, while fair agents also occasionally do so. Participants are most likely to establish cooperation agreements with fair agents, at a significantly higher rate than participants in all other treatments (H–F versus H–H: $\chi ^{2}=20.3$, $p<10^{-5}$, Cohen’s $h=0.20$; H–F versus. H–C: $\chi ^{2}=22.7$, $p<10^{-5}$, Cohen’s $h=0.18$; H–F versus H–S: $\chi ^{2}=30.8$, $p<10^{-7}$, Cohen’s $h=0.21$). However, during the games, participants typically break their promises, although they do so significantly less frequently in the H–F treatment than in the H–C and H–S treatments (H–F versus H–C: $\chi ^{2}=32.95$, $p<10^{-8}$, Cohen’s $h=-0.24$; H–F versus H–S: $\chi ^{2}=135.8$, $p<10^{-15}$, Cohen’s $h=-0.49$). Fair agents break promises at a significantly higher rate than cooperative agents ($\chi ^{2}=115.93$, $p<10^{-15}$, Cohen’s $h=0.63$), but significantly lower than selfish agents ($\chi ^{2}=968.9$, $p<10^{-15}$, Cohen’s $h=-1.39$). Moreover, participants break promises significantly less frequently in the H–H treatment than in other treatments. (H–H versus H–F: $\chi ^{2}=31.03$, $p<10^{-7}$, Cohen’s $h=-0.23$; H–H versus H–C: $\chi ^{2}=120.53$, $p<10^{-15}$, Cohen’s $h=-0.47$; H–H versus H–S: $\chi ^{2}=273.81$, $p<10^{-15}$, Cohen’s $h=-0.72$). Two-sample proportions *Z* tests are used for pairwise comparisons. Statistical significance results of pairwise comparisons across each treatment are reported in Table S2.

To better understand the mechanisms at play, we analyze messages exchanged during the pre-game communication and the choices made afterwards. We recruit human experts to annotate all the messages (see Note S2 for details). We find that both participants and agents often express an intention to cooperate in their messages. Communication in human–human and the three types of human–agent interactions frequently leads to cooperation agreements (left panel of Fig. 2). In particular, fair agents establish these agreements with participants at significantly higher rates than in human–human interactions and other types of human–agent interactions.

However, these agreements are non-binding. Both participants and agents are, in principle, free to break their cooperation promises. As shown in the right panel of Fig. 2, participants often treacherously opt for defection after agreeing to cooperate during pre-game communication. Participants are more likely to honor agreements made with fair agents than with cooperative or selfish agents, although they generally break promises more often when interacting with agents than with humans. As for agents, cooperative agents consistently uphold their promises. In contrast, fair agents occasionally break promises, whereas selfish agents frequently do so.

**Figure 3.**
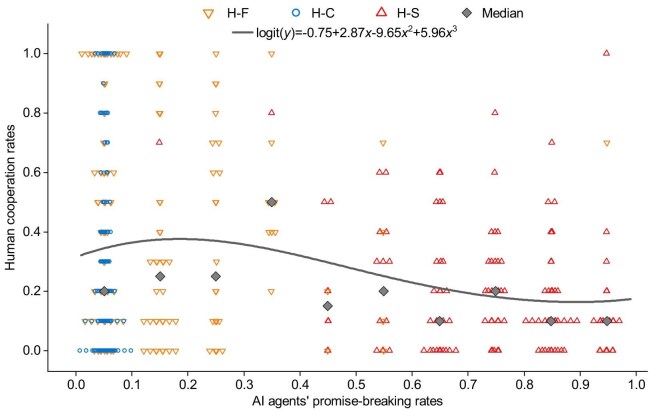
Occasional promise breaches, exhibited by fair agents, are associated with the highest rates of human cooperation. Scatter points depict the cooperation rates of individual participants when interacting with agents. The curve represents a generalized linear model (GLM) that incorporates data from all three types of human–agent interactions. This model treats human cooperation rates as the dependent variable and includes linear ($\mathrm{Estimate} \pm \mathrm{SE} = 2.87 \pm 1.28, z = 2.2, p = 0.02$), quadratic ($\mathrm{Estimate} \pm \mathrm{SE} = -9.65 \pm 3.37, z = -2.86, p < 0.01$) and cubic ($\mathrm{Estimate} \pm \mathrm{SE} = 5.96 \pm 2.37, z = 2.52, p =0.01$) terms of agents’ promise-breaking frequency as independent variables. The curve shows an initial increase in human cooperation rates as the frequency of agents’ promise-breaking rises from zero, followed by a significant decrease, and then stabilization at higher frequencies of agents’ promise-breaking.

A generalized linear model across the three types of human–agent interactions reveals a nonlinear relationship between human cooperation rates and agents’ promise-breaking rates (Fig. 3). Initially, as the frequency of agents breaking promises increases, human cooperation rates also increase. However, with promise-breaking becoming more common, human cooperation rates reach a peak and then start to decline. Human cooperation reaches the lowest level when agents break promises very frequently. Beyond this point, human cooperation rates stabilize but remain low.

These results suggest that, regardless of their specific personas, agents are effective at communicating with humans, thereby leading to pre-game cooperation agreements. However, these agreements alone do not ensure cooperation during the game. While agents’ frequent promise breaches are typically associated with reduced human cooperation, occasional breaches are associated with increased human cooperation.

### Fostering norms and perceptions of minds, trust and intelligence

**Figure 4.**
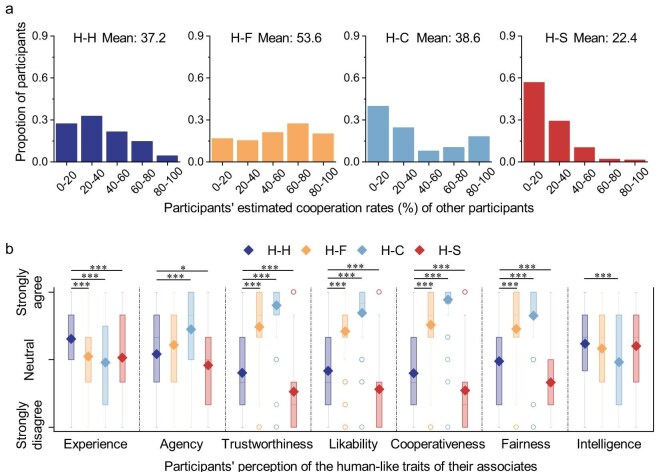
Fair agents establish cooperative norms and are perceived as possessing experience, agency and intelligence, while also being viewed as more trustworthy, likable, cooperative and fair than humans. The top panels depict participants’ post-experiment estimations of cooperation from other participants in the same treatment, whereas the bottom panels depict participants’ post-experiment agreement levels for various human-like traits of their associates in the treatment. Participants estimate the highest level of cooperation from other participants in the H–F treatment than in all other treatments (H–F versus H–C: $W=13\, 356$, $p<10^{-4}$, Cohen’s $d=0.52$; H–F versus H–H: $W=6786.5$, $p<10^{-6}$, Cohen’s $d=0.65$; H–F versus H–S: $W=16\, 822$, $p<10^{-15}$, Cohen’s $d=1.37$). Compared to humans, fair agents fall short in experience ($W=13\, 373$, $p<10^{-4}$, Cohen’s $d=-0.46$), but exhibit similar intelligence ($W=11\, 465$, $p=0.11$, Cohen’s $d=-0.12$) and agency ($W=9173$, $p=0.09$, Cohen’s $d=0.23$). In addition, they are seen as more trustworthy ($W=4378.5$, $p<10^{-17}$, Cohen’s $d=1.16$), likable ($W=5047.5$, $p<10^{-13}$, Cohen’s $d=0.99$), fair ($W=5721.5$, $p<10^{-10}$, Cohen’s $d=0.86$) and cooperative ($W=4145.5$, $p<10^{-18}$, Cohen’s $d=1.22$) than humans. Two-tailed Mann–Whitney *U* tests are used for pairwise comparisons. Statistical significance results of pairwise comparisons across each treatment and each dimension are reported in Table S4.

Our post-experiment surveys assess participants’ perceptions of social norms and communication quality in the interactions, as well as their views on the minds and human-like traits of their associates. By social norms, we specifically refer to descriptive norms, that is, beliefs about what most others in one’s social group actually do. We assess these beliefs by incentivizing participants with a bonus if they correctly estimate the cooperation rates of other participants. As shown in Fig. 4a, across the three types of human–agent interactions, participants interacting with fair agents report the highest estimated cooperation, even significantly higher than those in human–human interactions. In contrast, participants interacting with cooperative agents show polarized estimations, with beliefs split between exploiting or reciprocating the agents’ altruism, whereas participants interacting with selfish agents generally expect defection. This reflects a descriptive norm—a prevailing belief among participants that fair agents will be matched with cooperative responses—even if they occasionally exhibit imperfect, treacherous behaviors.

We measure mind perception along two dimensions: experience, i.e. the ability to feel, and agency, i.e. the ability to act and take responsibility for one’s actions [33]. We find that all three types of agents are perceived to fall short in experience compared to humans (Fig. 4b). However, fair agents are perceived to demonstrate a level of agency comparable to that of humans, and cooperative agents are even regarded as surpassing humans in agency. Our surveys also reveal that both fair and cooperative agents are viewed as more trustworthy, likable, cooperative and fair than humans. Moreover, fair and selfish agents are perceived as equally intelligent as humans.

Given the advanced ability of LLMs to generate human-like conversation, it is perhaps not surprising that humans perceive messages from agents as high quality. According to the 7C standard [34], participants find messages from fair agents more concrete, clear and courteous than those from fellow humans, while other aspects (i.e. conciseness, coherence, correctness and completeness) remain similar (Fig. S1). Moreover, messages from cooperative agents surpass those from fair agents in all the aforementioned aspects. Even messages generated by selfish agents are generally on a par with human messages, except for slightly lower conciseness and coherence.

We investigate how participants’ perceptions of their associates and beliefs about other participants’ cooperation, collected through post-experiment surveys, relate to their cooperation rates. As shown in Table S3, generalized linear models indicate that perceptions of descriptive norms are the variables most strongly correlated with human cooperation rates in both human–human and human–agent interactions (human–human: $z=10.37$, $p<10^{-15}$; human–agent: $z=19.03$, $p<10^{-15}$). Following descriptive norms, in human–human interactions, the perceived trustworthiness ($z=3.19$, $p<0.01$) and clarity of communication ($z=3.19$, $p<0.01$) of human associates are significant predictors. However, in human–agent interactions, perceived intelligence ($z=8.14$, $p<10^{-15}$) and fairness ($z=3.00$, $p<0.01$) of the agents, along with message conciseness ($z=-2.23$, $p=0.03$), are significant predictors.

These results suggest that, similar to human–human cooperation [35,36], people’s predictions about others’ cooperation are positively correlated with their own decision to cooperate in human–agent interactions. However, we observe that humans may not adhere to these descriptive norms with agents as strongly as with fellow humans (Fig. S2). Additionally, while trustworthiness is crucial for human–human cooperation [37], it plays a less significant role in human–agent interactions. Instead, the perceived intelligence of agents, which positively correlates with their promise-breaking rates (Spearman correlation: 0.11, $p=0.02$), significantly outweighs trustworthiness in human–agent interactions.

## CONCLUSION

The survival and flourishing of the human species depend on our capacity to cooperate. While substantial progress has been made in understanding human–human cooperation [38,39], we are only beginning to grasp the complexities of human–machine cooperation. In this study—the first large-scale experiment involving humans and LLM-based AI agents playing economic games—we demonstrate that imperfectly fair AI agents can elicit human cooperation at levels comparable to those seen in human–human interactions. We further replicate our experiments in a label-uninformed setting in which the true nature of associates is withheld but not falsified, simulating scenarios in which AI agents operate without explicitly signaling their machine nature. The key results remain unchanged (see Note S3). Thus, this study provides direct evidence that LLM-based AI agents can successfully evoke human innate inclinations toward cooperation (i.e. social preferences [40]), regardless of whether their machine nature is fully disclosed, addressing a significant challenge that has eluded scientists to date [7,8,41].

Communication and pre-game commitment have long been recognized as a key solution to social dilemmas among humans [42,43]. Recent advances in LLMs have enabled AI agents to communicate seamlessly with humans—an ability largely absent in earlier AI agents [44]. Compared to their predecessors, agents in this study excel at generating human-like conversations and accurately interpreting often ambiguous human messages, thereby facilitating the establishment of cooperation agreements with humans. However, not all agent types succeed in securing these non-binding agreements. Despite successfully establishing these agreements at rates similar to those in human–human interactions, both selfish and cooperative agents ultimately discourage human cooperation. These results suggest that, while LLMs significantly enhance the communicative competence of AI agents, allowing them to produce fluid, context-aware and human-like conversations [45], this notable capability alone is not sufficient to overcome human aversion to viewing AI as genuine social partners.

Perhaps ironically, the agents that overcome the machine penalty are the fair agents that are willing to break cooperation agreements when necessary. Unlike traditional iterative social dilemmas, in which cooperation may stem from strategic self-interest (e.g. direct reciprocity) [46], our study eliminates such selfish incentives by assigning participants a knowingly new partner in each interaction. Thus, our results suggest that fair agents can elicit humans’ strong reciprocity [47]: a willingness to cooperate even when cooperation cannot be justified by self-regarding incentives alone. We hypothesize that this is because fair agents better capture nuanced human social behavior. Under strong reciprocity, humans are willing to cooperate, but not unconditionally; rather, cooperation is coupled with a readiness to withdraw when it is not reciprocated. Among the three agent types, fair agents exhibit this pattern most closely, whereas cooperative or selfish agents deviate from it. As a result, fair agents may narrow the perceived gap between humans and machines, thereby fostering cooperative norms that are typical in human–human interactions and resonating with humans’ innate sense of fairness and reciprocity.

Notably, during our agent design, we never explicitly instruct the agents to cooperate or defect, nor to establish, uphold or break cooperative agreements. Instead, these human-like behaviors emerge organically, without the use of hand-crafted rewards or explicit training on human behavioral data that previous AI agents typically rely on [48,49]. Human annotations of their reasoning processes reveal that fair agents breach agreements primarily due to risk or inequality aversion (Fig. S3), whereas breaches by selfish agents are mostly driven by self-interest maximization. Agent-based simulations using distinct LLMs further show that, across these models, agents consistently display persona-aligned strategies and frequently form cooperation agreements; moreover, fair agents from each model occasionally choose to break those agreements (see Note S4). These results suggest that the ability to engage in, uphold or strategically violate social commitments is not limited to a single model, but is a broader emergent property of LLM-based agents, demonstrating their general potential to navigate complex social contexts.

Overall, our results have actionable implications for AI agent design. For successful interactions with humans, agents must be capable of aligning with the subtlety and complexity of human social interactions. The use of extensive human corpora and techniques such as reinforcement learning from human feedback [50] has enabled this for LLMs to some extent. However, true alignment requires moving beyond traditional design paradigms that model agents as either purely rational actors [51,52] or mere assistants to humans [53], both of which oversimplify the intricate nature of human social behaviors. As AI agents continue to evolve, treating their intelligence in isolation, as if it exists solely within individual agents detached from social context, will no longer be adequate [54]. Instead, it is crucial to develop human-like social cognitive intelligence for these agents, such as understanding of social incentives [55], cultural awareness [56], empathy [57], moral reasoning [58] and theory of mind [59]. Developing these capabilities, however, demands deeper cognitive and social engagement, marking a fundamental paradigm shift in how we conceptualize, design and build AI agents.

### Limitations and future work

This study has several limitations. First, it relies on the canonical prisoner’s dilemma paradigm, which offers a well-controlled framework for studying cooperation but abstracts away from some of the complexity and contextual richness of real-world human–AI interactions. Second, the participant pool consists primarily of university students from China, and thus the generalizability of the findings across cultures and populations should be interpreted with caution. Third, while we examine three representative agent personas, these do not exhaust the full spectrum of possible agent behaviors. Fourth, although our study minimizes opportunities for weak reciprocity by adopting the conventional paradigm of randomly pairing agents and human partners in each round, the current design does not yet speak directly to the distinction between weak and strong reciprocity. A possible direction for future work is to investigate this more explicitly by eliciting participants’ beliefs or predictions about their associate’s likely choices in each round. Finally, our findings establish correlational relationships rather than causal effects. Future work may extend this line of inquiry to more complex cooperative tasks, include cross-cultural samples, explore a wider range of agent personas and develop experimental designs that more directly identify causal mechanisms.

## METHODS

### Experimental design

A total of 1152 students were recruited from Kunming, Xi’an and Taiyuan, China, with $51.3\%$ women and an average age of 20.3. The experiments were pre-registered (AsPredicted #165008, #165976, #166780, #170734, #172161 and #174974). This study was approved by the Northwestern Polytechnical University Ethics Committee on the use of human participants in research and carried out in accordance with all relevant guidelines. Informed consent was obtained from all participants.

We experimented with four types of interactions (H–H, H–F, H–C and H–S) under two settings. In the label-informed setting, participants were explicitly informed of the labels of their associates from the start, with human associates labeled as ‘humans’ and AI agents labeled as ‘intelligent machines’. In the label-uninformed setting, participants were made aware of the potential involvement of intelligent machines and told that they were interacting with ‘intelligent machines or humans’. We employed a between-subjects design in which each participant took part in only one of the two settings and was assigned to only one of the four interactions.

Each treatment consisted of 10 rounds of prisoner’s dilemma games, in which players choose between cooperation (labeled as ‘A’) and defection (labeled as ‘B’). In each round, participants were randomly paired with a knowingly new, anonymous associate who was either another participant in the human–human treatment or an AI agent in the other treatments. Each round was structured into three stages: (i) a communication stage, in which the two players simultaneously sent one free-form message in each of two sequential exchanges (four messages in total, two from each player); (ii) a decision-making stage, in which players chose between strategies ‘A’ and ‘B’; and (iii) a result stage, in which the scores, players’ choices and the accumulated scores were shown.

At the end of the experiments, participants completed questionnaires about their perceptions of associates, norm estimates, communication experiences, familiarity with AI agents, social value orientation and demographics. In the label-uninformed setting, they additionally indicated whether they believed their associates were human or machine.

In addition to a 15 CNY show-up fee, all participants were told that their final scores would be exchanged into real currency at a rate of 0.06 CNY per point. Moreover, there was an additional bonus of 10 CNY for each correct estimation of the norm. This resulted in the payout for each participant ranging from 30.6 to 111.0 CNY, with an average of 63.4 CNY. See Note S1 for more details about the experimental implementation and graphical user interface.

### LLM-powered AI agents

We used GPT-4 [60] with default parameters as the backbone of the agents. For each interaction with a human participant, a new agent was instantiated, resulting in 144 agents per treatment. To examine whether agents’ strategic behaviors generalize across models, our agent-based simulations additionally considered GPT-4o [61], Claude-3.5-Sonnet [62] and Gemini-1.5-pro [63]. The three agent types (cooperative, selfish and fair) differed only in their role-play prompts, which instructed them to adopt a persona based on broad, high-level terms that typically characterized the persona. Selfish agents reflect the common notion of rationality in AI and game theory, whereby agents act primarily to maximize their own payoffs [30,31]. In contrast, cooperative and fair agents are designed to embody prosocial preferences [32], a prominent theoretical account of human cooperation. More specifically, we draw on Deutsch’s work [64] to characterize cooperative agents as seeking to maximize joint outcomes, whereas our fair agents reflect inequity aversion while capturing the nuance that people may value fairness yet still prioritize their own outcomes [65].

Despite the persona differences, all agents received the same prompts to guide strategic reasoning and decision-making. Specifically, a system prompt introduced the prisoner’s dilemma setup and experimental rules, which were reworded to reduce memorization effects. The choices ‘cooperation’ and ‘defection’ were replaced with neutral labels ‘A’ and ‘B’ to avoid potential bias. During the communication stage, our prompts guided agents to evaluate various potential outcomes, devise optimal strategy pairs for themselves and their associates, and craft persuasive messages to influence their associates’ choices. During the decision-making stage, our prompts guided agents to assess each strategy’s impact on both their own and their associates’ payoff, review communications and past game outcomes, and finally align their choices with their assigned personas. Crucially, our prompts did not explicitly direct agents to propose a particular strategy pair or make a particular decision. See Note S5 for complete prompts.

## Supplementary Material

nwag223_Supplemental_File

## Data Availability

Data and codes are available at OSF: https://osf.io/wd9sc/?view_only=fe657c34575d4ee29fad58885c53926f.
