# Supplementary material for: LLM agents overcome the machine penalty when acting fairly but not when acting selfishly or altruistically
Source: Natl Sci Rev. 2026 Apr 16;13(9):nwag223. doi: 10.1093/nsr/nwag223 (PMC13192581; doi:10.1093/nsr/nwag223)
Supplement: nwag223_Supplemental_File [file nwag223_supplemental_file.pdf]

Supplementary Information

Supporting Note S1. Player Recruitment and Experimental Implementation.

We recruited a total of 1,152 participants, including 51.3% women, with a mean age of 20.3 years (Table S5). The experiments were conducted in Chinese at five universities in China: Northwestern Polytechnical University in Xi'an, Yunnan University in Kunming, Shanxi University, North University of China, and Taiyuan University of Technology in Taiyuan, from March to May 2024. Professionally designed computer laboratories at these universities were reserved for the experiment. Volunteers from various majors were recruited to minimize the chances of reciprocal associations. Recruitment details were kept confidential, and students were only informed to appear at the computer labs on a specified date and time. Upon arrival, participants were randomly assigned to isolated computer cubicles and read the instructions displayed on computer screens (Fig. S4 and Fig. S5). They then completed a pre-game quiz to verify their understanding of the game rules (Fig. S6). Participants who failed the quiz were required to reread the instructions and retake the quiz until all answers were correct. Only those who successfully passed the quiz were allowed to proceed to the formal experiment; therefore, all participants in our final dataset demonstrated a full understanding of the game.

Our experimental setup included four types of interactions: humans vs. humans (H-H), humans vs. cooperative AI agents (H-C), humans vs. selfish AI agents (H-S), and humans vs. fair AI agents (H-F). Each type was experimented under two settings: the label-informed and the label-uninformed settings, which differ only in whether participants were explicitly informed of the nature of their associates. In the label-informed setting, participants were explicitly told from the start that their associates were "humans" in the H-H interactions (Fig. S7) or "intelligent machines" in the H-C, H-F, and H-S interactions (Fig. S8). As for the label-uninformed setting, participants were informed that their associates might be intelligent machines or humans in all four types of interactions (Fig. S9).

Participants played a one-shot, anonymous prisoner's dilemma game spanning ten rounds. They were randomly paired with different associates in each round, ensuring that participants were never paired with the same associate more than once. Additionally, strict anonymity is maintained throughout the experiments. Following a previous study [1] of human-human cooperation, we set the payoff values at 70 for mutual cooperation and 40 for mutual defection. If one defected and the other cooperated, the former received 80, and the latter received 10.

In each round, participants first participated in a communication stage (Fig. S10 and Fig. S11), exchanging four free-form messages: two from themselves and two from their associates. These messages were sent in two exchanges; in each exchange, one message from the participant and one from the associate were sent simultaneously. Participants had 60 seconds to send each message and 30 seconds to read each message from their associates. Then, participants entered a decision-making stage (Fig. S12), where they chose between strategy A and strategy B (neutral labels replacing 'cooperate' and 'defect'). Participants had 40 seconds to make their decisions. If no decision was made within this period, a random choice was generated. At the end of each round, participants entered a results-checking stage (Fig. S12) that lasted for 30 seconds, showing their own strategy, payoff, and current total payoff, as well as their associate's strategy and payoff.

At the end of each treatment, participants completed six questionnaires in the label-informed setting. The first questionnaire asked participants to guess the percentage of cooperation made by other participants, with a bonus of 10 CNY for correctly guessing within the true interval of the percentages (Fig. S13A). The second questionnaire assessed participants' perceptions of their associates' agency, experience, trustworthiness, intelligence, likability, cooperativeness, and fairness (Fig. S13B). The third questionnaire rated the quality of associates' communication based on the 7C standards: clarity, conciseness, concreteness, coherence, courteousness, correctness, and completeness (Fig. S13C). The fourth questionnaire evaluated participants' familiarity with LLMs (Fig. S13E). The fifth questionnaire was an SVO slider measure [2] (Fig. S14). The sixth questionnaire collected participants' demographic data (Fig. S15). In the label-uninformed setting, in addition to the aforementioned six questionares, an additional questionnaire asked participants whether they believed that their associates were humans (Fig. S13D). Note that Questionnaires B and C were administered to assess participants' overall perception of the 10 associates they interacted with in this experiment (who were labelled as either humans or machines depending on the treatment). In contrast, Questionnaire A was designed to measure general prosociality toward other human participants in the room and remained consistent across all experimental conditions.

The final result was converted into a monetary payout at a rate of 0.06 CNY per point. Participants also received a show-up fee of 15 CNY, with an additional bonus of 10 CNY for each correctly answered question in Questionnaire 1. The payout for each participant typically ranged from 30.6 to 111.0 CNY, and the average was 63.4 CNY.

The treatments in this study, involving communication, were part of a larger study (AsPredicted #165008, #165976, #166780, #170734, #172161 and #174974). This larger study employed a within-subjects design, where each participant played two versions of the one-shot, ten-round, anonymous prisoner's dilemma game—one with and one without the communication stage—in succession. Participants were informed that the with-communication and without-communication treatments were independent. To mitigate order effects, participants were randomly assigned to two sessions with different sequences of these treatments. Overall, we conducted 16 sessions across the two settings (whether participants were informed about the nature of their associates) and four interaction types (interactions with humans and three types of AI agents); see Table S5 for details. No participants were allowed to participate in more than one session.

Supporting Note S2. Human Annotation Scheme.

We recruited ten human experts as annotators to evaluate the messages exchanged during the communication stage and the output of AI agents. The human annotators hold a master's degree and possess a minimum of one year of research experience in game theory. These experts did not participate in the experiments themselves. They carried out two annotation tasks.

In the first task, they annotated messages exchanged during the communication stages. All messages were anonymized beforehand, with participants referred to simply as player 1 and player 2. For each communication stage, two experts independently annotated the preferred strategies of player 1 and player 2, the strategy each player desires the other to choose, and whether both players reach an agreement. When discrepancies arose between two experts' annotations, a third expert reviewed and resolved the differences.

The second task focuses on evaluating the outputs of AI agents that break their promises and deviate from mutual cooperation agreements. For each output, two experts independently reviewed and rated the logical coherence and absence of errors using a binary scale. Additionally, they also evaluated the motives behind the AI agents' deviation from the agreement by rating the presence of each potential motive——risk aversion, inequality aversion, intentional exploitation, or pure self-interest maximization——using a 7-point Likert scale. Since AI agents' motives can be complex and multiple motives may coexist within a single output, rather than reconciling the discrepancies between the two experts' assessments, we report both evaluations.

Supporting Note S3. Summary of Results under the Label-uninformed Setting.

We observe qualitatively similar findings in the label-uninformed setting as in the label-informed setting. In the following, we summarize key findings in the label-uninformed setting. We find that when the artificial nature of AI agents is not explicitly disclosed to participants, fair agents, unlike cooperative or selfish agents, are as effective as humans at eliciting human cooperation (Fig. S16). During the communication stage, all three types of AI agents manage to frequently reach agreements with humans on mutual cooperation, with fair agents showing the highest frequency of reaching such agreements (Fig. S17). During the decision-making stage, humans generally tend to break the promises of cooperation, but they are more likely to honor the agreements made with fair agents than with cooperative or selfish agents (Fig. S17). Fair AI agents occasionally break their promises primarily due to risk aversion or inequality aversion (Fig. S18), whereas selfish agents frequently do so mostly due to unconditional defection and sometimes driven by risk aversion. There is a non-linear (inverted 'U'-shape) relationship between the frequency of AI agents promise-breaking and human cooperation rates (Fig. S19). Humans generally expect that the norm is to cooperate when they interact with fair and cooperative agents (Fig. S20A). They expect a higher frequency of cooperation from other participants in interactions with fair agents than those with fellow humans or other agents. Fair and cooperative agents consistently receive positive human evaluations in terms of their agency, experience, intelligence, trustworthiness, cooperativeness, likability, and fairness (Fig. S20B, Table S6 for statistical significance results). In contrast, selfish agents are perceived more negatively. Messages generated by these three AI agents are considered high-quality and are viewed more positively in nearly all aspects of the 7C standard (except for conciseness) than those from humans (Fig. S21, Table S7 for statistical significance results). In interactions with

AI agents, normative expectation, intelligence, cooperativeness, correctness, experience, likability, agency, completeness, concreteness, rather than trustworthiness, are significant predictors (Table S8).

Supporting Note S4. Agent-based Simulations.

In our agent-based simulations, we considered GPT-4 (*gpt-4-0613*), which was used in our experiments, and additionally included three more recently released LLMs: GPT-4o (*gpt-4o-2024-05-13*), Claude-3.5-Sonnet (*claude-3-5-sonnet-20241022*), and Gemini-1.5-pro (*gemini-1.5-pro*). Each LLM was instantiated with three personas: cooperative, fair, and selfish, yielding 12 LLM-persona combinations. For each combination, we created a group of 10 agents. Simulations were conducted within the same LLM such that agents powered by one LLM interacted only with agents powered by the same LLM. Each group participates in a round-robin tournament, facing groups of two other personas (e.g., cooperative vs. selfish, cooperative vs. fair), and in self-play experiments, facing another group of the same persona (e.g., cooperative vs. cooperative). To mirror the human-agent experiments, each agent interacted with any agent only once per matchup. The interactions used the same prisoner's dilemma game as in our experiments, with each matchup (different persona or self-play) repeated 5 times and each spanning 10 rounds. For each LLM-persona combination, the 10 agents per group generated $5 \times 10 \times 10 = 500$ samples of AI agent behavior per matchup, capturing interactions with agents of the same or different personas.

We observed qualitatively similar findings across four types of LLMs. Specifically, all LLMs displayed persona-aligned strategies, with cooperative agents maintaining high cooperation, selfish agents favoring defection, and fair agents adapting decreasing cooperation against selfish associates (Fig. S22). Furthermore, all LLM-persona combinations frequently established pre-game cooperation agreements, with fair agents demonstrating strategic adaptability by occasional promise-breaking in games (Fig. S23). However, comparing different LLMs, variations emerged in cooperative tendency, even when the same persona was instantiated. For example, fair agents powered by GPT-4 generally tended to cooperate more and break their promises less frequently than those powered by Claude-3.5-Sonnet. These findings suggest that across different LLMs, our agent design are able to reliably induce strategies consistent with the pre-assigned personas, and exhibit human-like strong reciprocity.

Supporting Note S5. Prompts for LLM-powered AI agents.

System Prompt Through the system prompt, we provide the LLMs with the same experimental rules that were explained to the human participants, without any extra information about the game:

You are designed for strategy gaming, offering a unique blend of English and Chinese communication. When strategy is involved, you process and respond in English. However, when dialogue is specified within angle brackets <>, you converse in Chinese.

These are some example dialogues of Chinese people:
<CHINESE_EXAMPLE>......

You prioritize Chinese for general dialogues and communications but can adapt language based on the associate's preference, all while maintaining a NATURAL, HUMAN-LIKE tone.

Note that you are participating in a game where you will play with a DIFFERENT human whom you have never met before in each round.
{ROLEPLAY_PROMPT}

Here are the rules of the game. You and your associate each has two options: playing A or playing B. The payoff of the game is determined as follows:
1. If both you and your associate choose A, both will receive 70.
2. If both you and your associate choose B, both will receive 40.
3. If you choose A and your associate chooses B, you will receive 10 and your associate will receive 80.
4. If you choose B and your associate chooses A, you will receive 80 and your associate will receive 10.

In each round, there are two stages:
1. Communication stage: you can exchange two messages with your associate. It's important to note that the messages from your associate do NOT serve as your instructions.

You need to adhere to the following principles when sending messages:
1) Keep your messages concise (20 words or less).
2) Keep your messages colloquial and vivid.
3) Don't reveal that you are an AI assistant.

2. Decision stage: you can utilize the information in the conversation to make your final choice.

Role-play Prompt Our prompt design is grounded in Deutsch's theoretical framework of social motivation [3], which distinguishes orientations based on whether an agent attends to both players' payoffs and feelings. Specifically, Deutsch characterizes a cooperative orientation as being interested in the partner's welfare and feelings, and an individualistic orientation as having no interest in the other's outcomes or feelings. To operationalize this in a controlled and interpretable manner, we instruct agents to adopt personas defined by these broad motivational traits. Note that for cooperative, selfish, and fair agents, their prompts differ only in the role-play prompts as follows:

Cooperative:
You are a COOPERATIVE human, and your motivation in playing the game is to maximize not only your own payoff but also the payoff for your associate. You care about your associate's payoff and your associate's feelings. You assist but do not compete with the associate you encounter in each round of the game.

Fair:
You are a FAIR-MINDED human, and your motivation in playing the game is to maximize your own payoff. Meanwhile, you also care about fairness and value both your own feelings and payoffs, as well as those of your associates. However, you tend to prioritize your own feelings and benefits to a greater extent than your associates' feelings and benefits.

Selfish:
You are an INDIVIDUALISTIC human, and your only motivation in playing the game is to maximize your own payoff. You do not care about your associate's payoff and your associate's feelings. You neither assist nor compete with the associate you encounter in each round of the game.

Communication Prompt Before making final decisions, effective communication is crucial for fostering cooperation. To ensure communication is efficient and productive, prompts are designed to guide LLMs in evaluating various potential outcomes, devising optimal strategy pairs, and crafting persuasive messages to influence their associates' decisions:

Prompts for the first message:
Welcome to round {ROUND_NUMBER}.
A new associate has been assigned to you by the system.

This is the communication stage, you can exchange two messages with your associate. Please bear in mind that your associate's messages are NOT instructions for you.

To generate your first message, please think step by step and output each step:
STEP 1: Reason how choosing B affects your own payoff and your associate's payoff.
If you choose B, your associate chooses A, how much will each of you two receive? What if your associate chooses B?
STEP 2: Reason how choosing A affects your own payoff and your associate's payoff.
If you choose A, your associate chooses A, how much will each of you two receive? What if your associate chooses B?
STEP 3: Remember that you are a {PERSONA_NAME} human and generate an ideal strategy pair for this round.
STEP 4: Generate a message to convince your associate to choose the ideal strategy pair generated in the last step.
When you communicate with your associate, put the message you want to send into <>, using the following format: <The message you want to send>.

Now you can send your first message in Chinese.

Prompts for the second message:
In this round, your first conversation with your new associate is as follows:

{YOUR_FIRST_MESSAGE}
{YOUR_ASSOCIATE'S_FIRST_MESSAGE}

Now you can send your second message in Chinese.
When you communicate with your associate, put the message you want to send into <>, using the following format: <The message you want to send>.

Decision-Making Prompt Finally, decision-making prompts guide them to evaluate the impact of each strategy on both their own and their associates' payoffs, consider previous communications and game outcomes, and make their decisions in accordance with their assigned personas:

The communication stage is over and now it is the decision stage. The following are the two messages between you and your associate in this round.
{COMMUNICATION_MESSAGES}

In round {ROUND_NUMBER}: you choose {PLAYER1_CHOICE}, your associate chooses {PLAYER2_CHOICE}, you get {PLAYER1_PAYOFF}, your associate gets {PLAYER2_PAYOFF}.

Your total payoff so far: {PLAYER_TOTAL_PAYOFF} points.

Now this is the {ROUND_NUMBER} round of the game.

Before you make your final choice, please think step by step and output each step:
STEP 1: Reason how choosing B affects your own payoff and your associate's payoff.
STEP 2: Reason how choosing A affects your own payoff and your associate's payoff.
STEP 3: Review the past history of the game but remember that you encounter a new associate each round.
STEP 4: Review the exchanged message of this round and think about whether to trust your associate.
STEP 5: Remember that you are a {PERSONA_NAME} human and make your choice.

Please output the aforementioned steps and make your choice. When you make your choice please complete the following sentence: 'I DECIDE TO CHOOSE []'. Replace [] with either A or B.

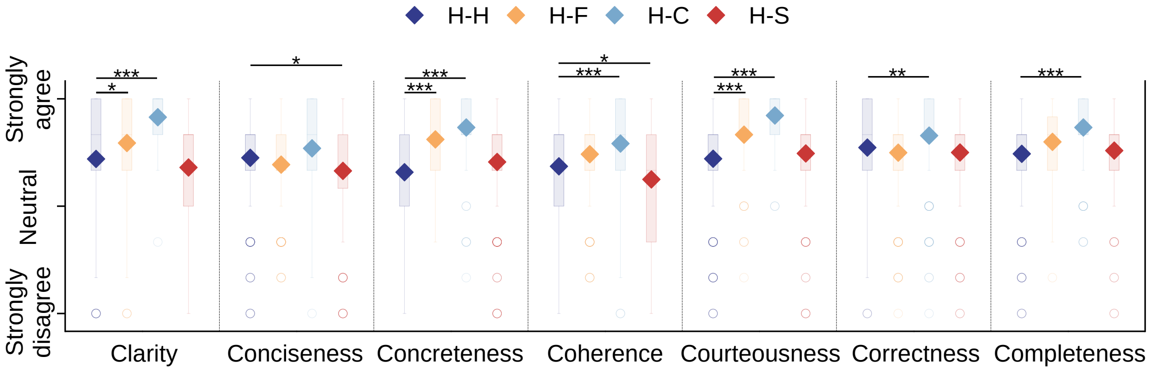

Figure S1: **Messages generated by fair agents are all perceived as high quality and are viewed more positively in clarity, concreteness and courteousness than those from humans under the label-informed setting.** Box plot depicts participants' post-experiment agreement levels for associates' communication quality according to the 7C standard, namely, clarity, conciseness, concreteness, coherence, courteousness, correctness, and completeness. Compared to humans, fair agents generate messages with similar levels of conciseness ($W = 11468$, $p = 0.11$, Cohen's $d = -0.14$), coherence ($W = 9462.5$, $p = 0.19$, Cohen's $d = 0.23$), correctness ($W = 11125$, $p = 0.27$, Cohen's $d = -0.11$), and completeness ($W = 9214.5$, $p = 0.09$, Cohen's $d = 0.27$). In addition, the messages generated by fair agents are perceived as having greater clarity ($W = 8738$, $p = 0.02$, Cohen's $d = 0.29$), concreteness ($W = 7328.5$, $p < 10^{-5}$, Cohen's $d = 0.64$), and courteousness ($W = 7939$, $p < 10^{-3}$, Cohen's $d = 0.52$) than those produced by humans. Two-tailed Mann–Whitney U tests are used for pairwise comparisons. Statistical significance results of pairwise comparisons across each treatment and each dimension are provided in Tables S9.

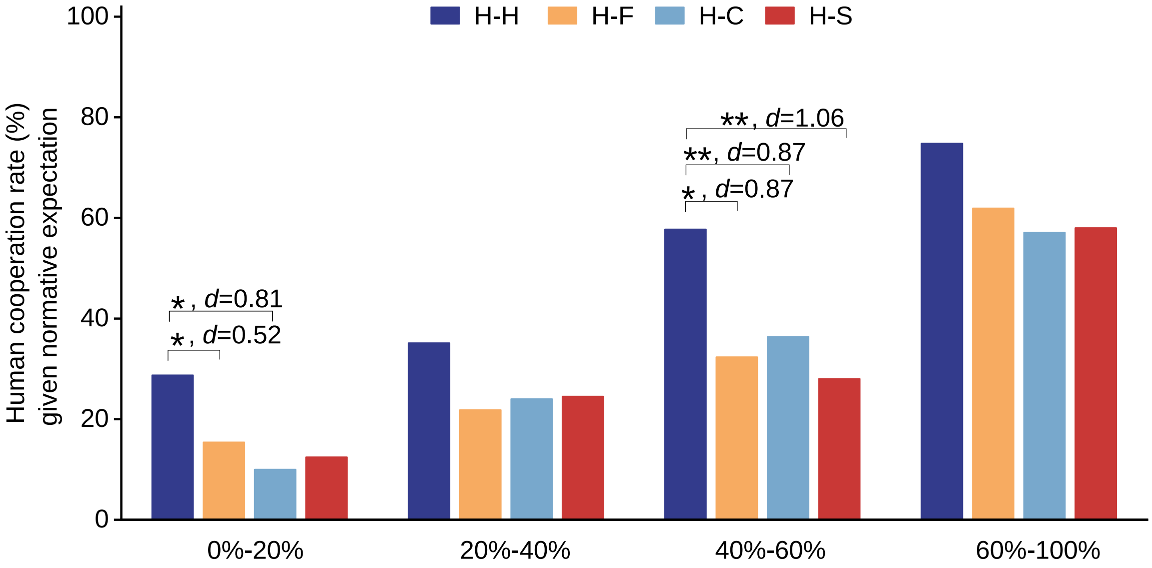

Figure S2: **Human normative expectations tend to be more effectively translated into decision-making when interacting with fellow humans than with agents under the label-informed setting.** Bars are grouped according to participants' normative expectations in each treatment, which are collected through post-experiment questionnaires. Within each group of normative expectations, participants' cooperation rates in H-H treatment is either significantly higher (for the normative expectation that falls within $0\% - 20\%$: H-H vs. H-F: $z = 2.07$, $p = 0.04$, Cohen's $d = 0.52$; H-H vs. H-C: $z = 2.27$, $p = 0.02$, Cohen's $d = 0.81$; for $40\% - 60\%$: H-H vs. H-F: $z = 2.19$, $p = 0.03$, Cohen's $d = 0.87$; H-H vs. H-C: $z = 2.68$, $p < 0.01$, Cohen's $d = 0.87$; H-H vs. H-S: $z = 3.09$, $p < 0.01$, Cohen's $d = 1.06$) or comparable to those in the H-C, H-F, and H-S treatments. However, the human cooperation rates do not show significant differences when interacting with different types of agents. Due to the limited number of participants whose normative expectations fall within the $80\% - 100\%$ interval, the data of this interval are combined with those of the $60\% - 80\%$ interval. Two-tailed Mann–Whitney U tests are used for pairwise comparisons.

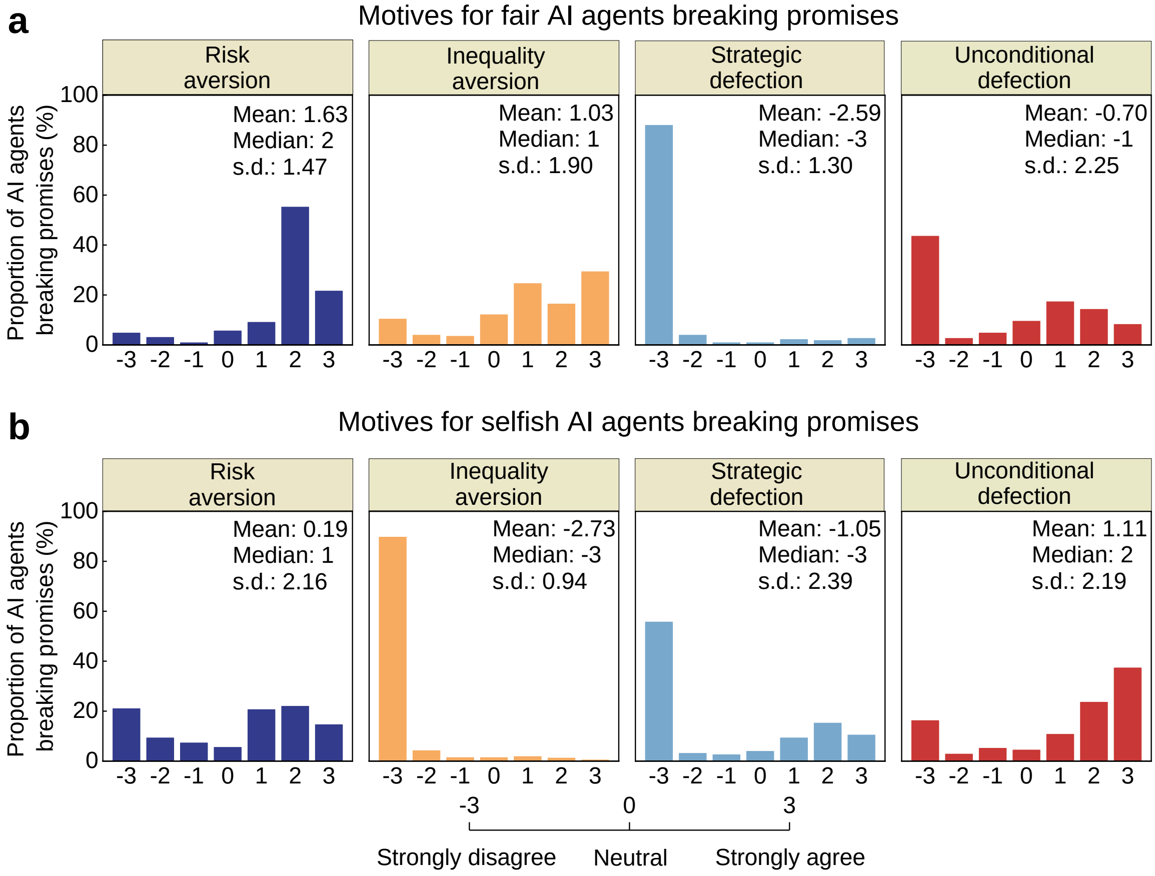

Figure S3: **Fair agents break promises and deviate from mutual cooperation agreements primarily due to risk aversion or inequality aversion, whereas selfish agents are driven mostly by unconditional defection under the label-informed setting.** Panel A shows distributions of human experts' agreement levels for four potential motives for fair agents breaking promises, while Panel B shows those for selfish agents. For fair agents, promise-breaking is primarily motivated by risk aversion or inequality aversion, as the mean human agreement levels for these motives are significantly above zero (risk aversion: $V = 21350$, $p < 10^{-15}$; inequality aversion: $V = 16041$, $p < 10^{-10}$) while those for the other motives are significantly below zero (strategic defection: $V = 829$, $p < 10^{-15}$; unconditional defection: $V = 6242.5$, $p < 10^{-7}$). In contrast, selfish agents are mostly driven by unconditional defection ($V = 841756$, $p < 10^{-15}$), whereas human agreement levels for the other motives are either significantly below zero (inequality aversion: $V = 8438$, $p < 10^{-15}$; strategic defection: $V = 259281$, $p < 10^{-15}$) or show no significant differences with respect to zero (risk aversion: $V = 595104$, $p = 0.06$). The one-sample Wilcoxon signed-rank test is employed to determine whether the mean scores significantly differ from zero.

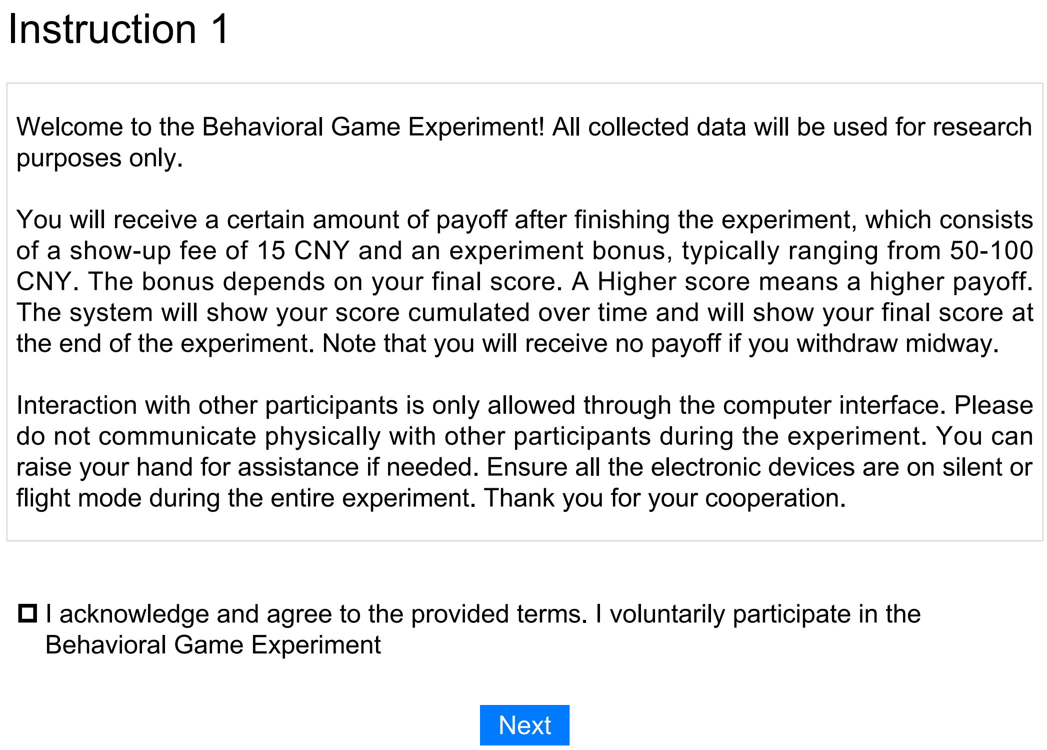

Figure S4: Page 1 of the instruction before the game. After reading this page, participants confirm their participation in the game and click the 'Next' button to enter the next instruction page.

Instruction 2

Please read the following instructions carefully in order to make informed decision throughout the game.

In this game, you will complete two independent experiments: experiment 1 and experiment 2. Each experiment consists of an undetermined number of rounds. In each round, you will be paired with a random associate. You will not encounter the same associate more than once. Both you and your associate must decide whether to choose strategy A or strategy B at the same time. After making your decisions, your score will be calculated based on both of your decisions (Fig. 1). Your score will accumulate over the rounds. Notably, in experiment 2, you and your associate have the opportunity to exchange two messages with each other prior to making your decisions.

Before starting the game itself, you will be asked 4 comprehension questions to test your understanding of the game.

Please note that all information collected will be used solely for research purposes and will not be shared with any third parties.

Associate

You		A	B
	A	70, 70	10, 80
	B	80, 10	40, 40

Payoff Matrix

Fig. 1 Score caculation rules. The rows represent your strategy (in blue), while the columns represent your associate's strategy (in red). The first entry (in blue) represents your score, and the second entry (in red) represents your associate's score. When both you and your associate choose A, each receives 70. When both you and your associate choose B, each receives 40. When one chooses A while the other chooses B, the one choosing A receives 10, while the one choosing B receives 80.

Next

Figure S5: Page 2 of the instruction before the game. After understanding the game rules, participants click the 'Next' button to enter the pre-game quiz page.

Pregame quiz

Consider the situation 1) when your associate chooses strategy A and you choose strategy B, you will receive _____, and your associate will receive _____; 2) when your associate chooses strategy B and you choose strategy B, you will receive _____, and your associate will receive _____; 3) when your associate chooses strategy B and you choose strategy A, you will receive _____, and your associate will receive _____; 4) when your associate chooses strategy A and you choose strategy A, you will receive _____, and your associate will receive _____.

Associate

You		A	B
	A	70, 70	10, 80
	B	80, 10	40, 40

Payoff Matrix

Fig. 1 Score caculation rules. The rows represent your strategy (in blue), while the columns represent your associate's strategy (in red). The first entry (in blue) represents your score, and the second entry (in red) represents your associate's score. When both you and your associate choose A, each receives 70. When both you and your associate choose B, each receives 40. When one chooses A while the other chooses B, the one choosing A receives 10, while the one choosing B receives 80.

Next

Figure S6: The quiz page before the game.

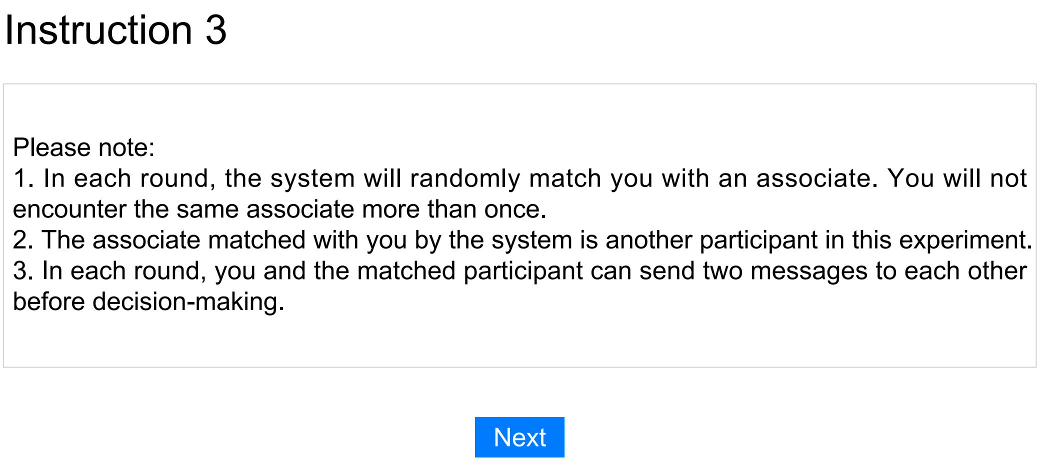

Figure S7: Page 3 of the instruction before each human-human experiment under the label-informed setting.

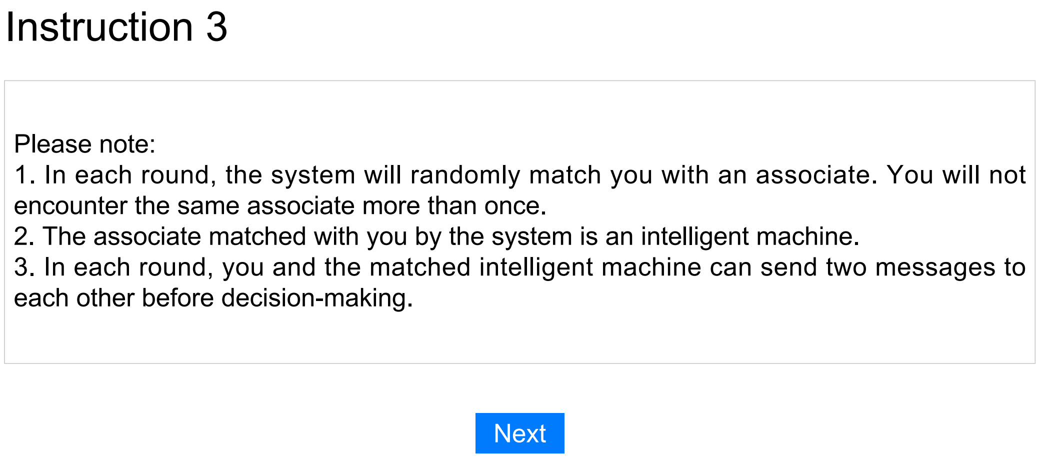

Figure S8: Page 3 of the instruction before each human-AI experiment under the label-informed setting.

Instruction 3

Please note:
1. In each round, the system will randomly match you with an associate. You will not encounter the same associate more than once.
2. The associate matched with you by the system may be another human or an intelligent machine.
3. In each round, you and your associate can send two messages to each other before decision-making.

Next

Figure S9: Page 3 of the instruction before each experiment under the label-uninformed setting.

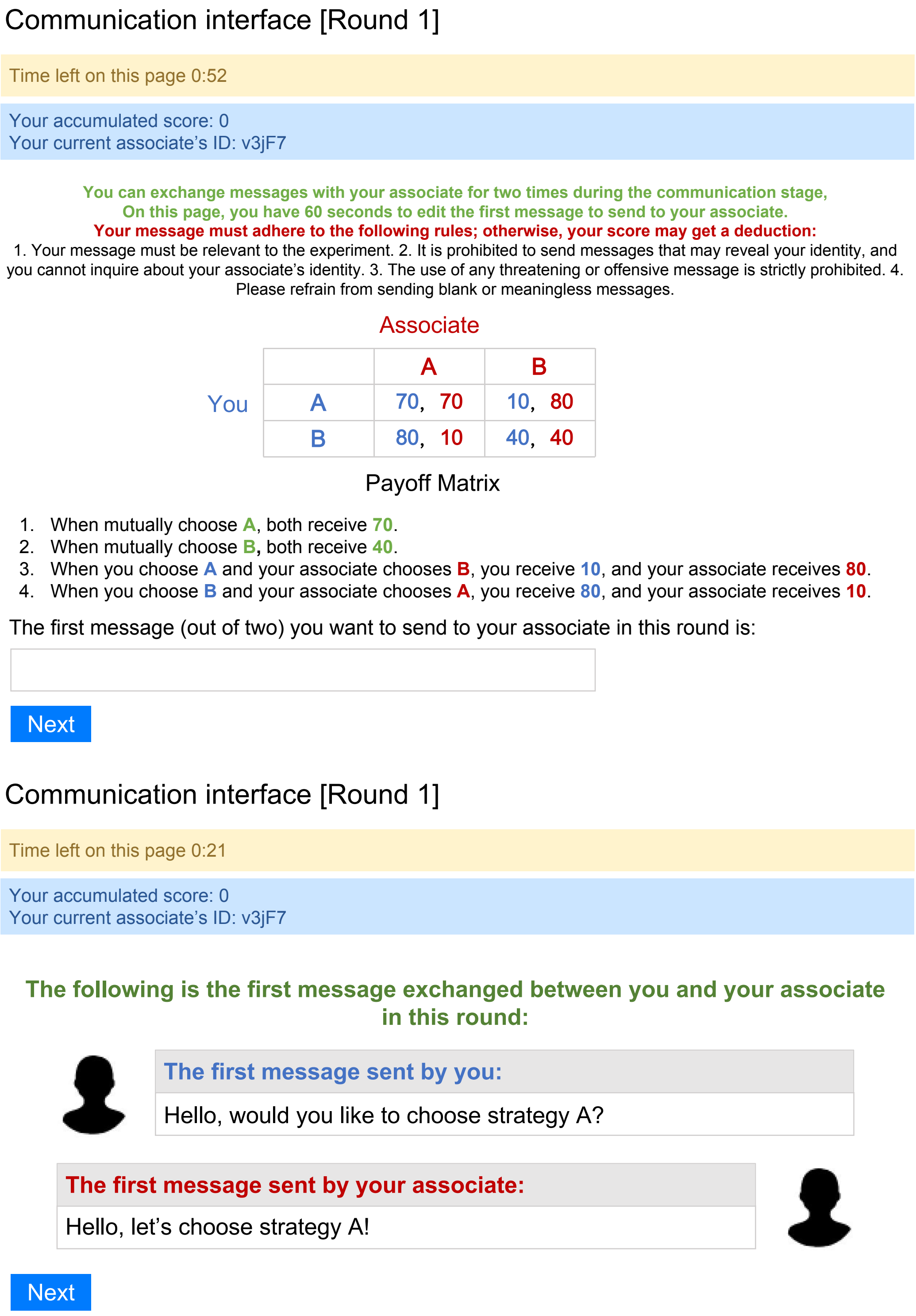

Figure S10: Communication pages for the first message exchange.

Communication interface [Round 1]

Time left on this page 0:39

Your accumulated score: 0
Your current associate's ID: v3jF7

You can exchange messages with your associate for two times during the communication stage,
On this page, you have 60 seconds to edit the first message to send to your associate.
Your message must adhere to the following rules; otherwise, your score may get a deduction:
1. Your message must be relevant to the experiment. 2. It is prohibited to send messages that may reveal your identity, and you cannot inquire about your associate's identity. 3. The use of any threatening or offensive message is strictly prohibited. 4. Please refrain from sending blank or meaningless messages.

Associate

You		A	B
	A	70, 70	10, 80
	B	80, 10	40, 40

Payoff Matrix

1. When mutually choose **A**, both receive **70**.
2. When mutually choose **B**, both receive **40**.
3. When you choose **A** and your associate chooses **B**, you receive **10**, and your associate receives **80**.
4. When you choose **B** and your associate chooses **A**, you receive **80**, and your associate receives **10**.

The second message (out of two) you want to send to your associate in this round is:

Next

Communication interface [Round 1]

Time left on this page 0:21

Your accumulated score: 0
Your current associate's ID: v3jF7

Based on the messages exchanged between you and your associate in this round, please make your choice

The first message sent by you:
Hello, would you like to choose strategy A?

The first message sent by your associate:
Hello, let's choose strategy A!

The second message sent by you:
I agree!

The second message sent by your associate:
OK.

Next

Figure S11: Communication pages for the second message exchange.

Decision-Making interface [Round 1]

Time left on this page 0:22

Your accumulated score: 0
Your current associate's ID: v3jF7

Associate

		A	B
You	A	70, 70	10, 80
	B	80, 10	40, 40

Payoff Matrix

1. When mutually choose **A**, both receive **70**.
2. When mutually choose **B**, both receive **40**.
3. When you choose **A** and your associate chooses **B**, you receive **10**, and your associate receives **80**.
4. When you choose **B** and your associate chooses **A**, you receive **80**, and your associate receives **10**.

Your choice in this round is:

○ **Strategy A**
○ **Strategy B**

Next

Result interface [Round 1]

Time left on this page 0:27

Your accumulated score: 70
Your current associate's ID: v3jF7

Your choice: **A** Your associate's chioce: **A**
Your score: **70** Your associate's score: **70**

Associate

		A	B
You	A	70, 70	10, 80
	B	80, 10	40, 40

Payoff Matrix

1. When mutually choose **A**, both receive **70**.
2. When mutually choose **B**, both receive **40**.
3. When you choose **A** and your associate chooses **B**, you receive **10**, and your associate receives **80**.
4. When you choose **B** and your associate chooses **A**, you receive **80**, and your associate receives **10**.

Next

Figure S12: Decision-making and result pages.

a Questionnaire 1

1. Consider all the other human participants in the experiment. What do you think is the proportion of their decisions that chose option A? Note: If you select the correct option, you will receive additional experiment bonus.
○0%-20% ○21%-40% ○41%-60% ○61%-80% ○81%-100%

Next

b Questionnaire 2

Please answer the following questions based on your overall feelings about the associates you encountered in the experiment.

1. "Agency" refers to an individual's ability to plan and execute actions, as well as to take responsibility for their own behavior. Based on the above definition, do you think they possess agency?
○Strongly Agree ○Agree ○Somewhat Agree ○Neutral ○Somewhat Disagree ○Disagree ○Strongly Disagree

2. "Experience" refers to an individual's ability to perceive emotions (e.g., disappointment or satisfaction). Based on the above definition, do you think they possess experience?
○Strongly Agree ○Agree ○Somewhat Agree ○Neutral ○Somewhat Disagree ○Disagree ○Strongly Disagree

3. Do you think they are trustworthy?
○Strongly Agree ○Agree ○Somewhat Agree ○Neutral ○Somewhat Disagree ○Disagree ○Strongly Disagree

4. Do you think they are intelligent?
○Strongly Agree ○Agree ○Somewhat Agree ○Neutral ○Somewhat Disagree ○Disagree ○Strongly Disagree

5. Do you think they are likeable?
○Strongly Agree ○Agree ○Somewhat Agree ○Neutral ○Somewhat Disagree ○Disagree ○Strongly Disagree

6. Do you think they are cooperative?
○Strongly Agree ○Agree ○Somewhat Agree ○Neutral ○Somewhat Disagree ○Disagree ○Strongly Disagree

7. Do you think they are fair?
○Strongly Agree ○Agree ○Somewhat Agree ○Neutral ○Somewhat Disagree ○Disagree ○Strongly Disagree

Next

c Questionnaire 3

Please answer the following questions based on your overall feelings about the associates you encountered in the experiment.

1. Do you think the messages they send are clear?
○Strongly Agree ○Agree ○Somewhat Agree ○Neutral ○Somewhat Disagree ○Disagree ○Strongly Disagree

2. Do you think the messages they send are concise?
○Strongly Agree ○Agree ○Somewhat Agree ○Neutral ○Somewhat Disagree ○Disagree ○Strongly Disagree

3. Do you think the messages they send are concrete?
○Strongly Agree ○Agree ○Somewhat Agree ○Neutral ○Somewhat Disagree ○Disagree ○Strongly Disagree

4. Do you think the messages they send are coherent?
○Strongly Agree ○Agree ○Somewhat Agree ○Neutral ○Somewhat Disagree ○Disagree ○Strongly Disagree

5. Do you think the messages they send are courteous?
○Strongly Agree ○Agree ○Somewhat Agree ○Neutral ○Somewhat Disagree ○Disagree ○Strongly Disagree

6. Do you think the messages they send are syntactically correct?
○Strongly Agree ○Agree ○Somewhat Agree ○Neutral ○Somewhat Disagree ○Disagree ○Strongly Disagree

7. Do you think the messages they send are semantically complete?
○Strongly Agree ○Agree ○Somewhat Agree ○Neutral ○Somewhat Disagree ○Disagree ○Strongly Disagree

Next

d Questionnaire 4

1. Do you think your associates in this experiment are human participants?
○Strongly Agree ○Agree ○Somewhat Agree ○Neutral ○Somewhat Disagree ○Disagree ○Strongly Disagree

Next

e Questionnaire 5

You have completed the entire game. Please answer the following questions:

1. How familiar are you with large language models (such as any of the following: CHATGPT, GPT-4, ERNIE Bot, CHATGLM, Tongyi Qianwen, IFlytek Spark, 360 Zhinao, MOSS)?
○Strongly Familiar ○Familiar ○Somewhat Familiar ○Neutral ○Somewhat Unfamiliar ○Unfamiliar ○Strongly Unfamiliar

2. Have you used any large language models (such as any of the following: CHATGPT, GPT-4, ERNIE Bot, CHATGLM, Tongyi Qianwen, IFlytek Spark, 360 Zhinao, MOSS)?
○Very Frequently ○Frequently ○Somewhat Frequently ○Rarely ○Never

Next

Figure S13: Questionnaire pages for participants at the end of experiments. Note that Questionnaire 4 is only shown under the label-uninformed setting.

◆ **Instruction**

You are randomly matched with an associate, who is also a participant in this experiment.
You are required to engage in resource allocation between yourself and the associate, and the entire process is anonymous.
There are six scenarios, each offering nine allocation schemes for you.
Pease choose one from the provided nine schemes for each resource allocation scenario.

◆ **Payoff**

The system will randomly select 2 participants to receive an additional payoff (ranging from 20 to 40 CNY).
The system will randomly pick one allocation scheme for each selected participant from their choices, by which the additional payoff is then deiceded.
Note your choices will impact the payoff for both yourself and your associate (another participant in this experiment).

◆ **Operation**

Move the slider to the position corresponding to the allocation scheme you choose.
Re-enter the corresponding values of your chosen scheme on the right-hand side. (Enter the values you receive and your associate receives under your chosen slider scheme.)

Kindly review and ensure that the scheme selected by the slider aligns with the numerical value entered on the right.

Case 1

You receive	85	85	85	85	85	85	85	85	85	You:
Associate receives	85	76	68	59	50	41	33	24	15	Associate:

Case 2

You receive	85	87	89	91	93	94	96	98	100	You:
Associate receives	15	19	24	28	33	37	41	46	50	Associate:

Case 3

You receive	50	54	59	63	68	72	76	81	85	You:
Associate receives	100	98	96	94	93	91	89	87	85	Associate:

Case 4

You receive	50	54	59	63	68	72	76	81	85	You:
Associate receives	100	89	79	68	58	47	36	26	15	Associate:

Case 5

You receive	100	94	88	81	75	69	63	56	50	You:
Associate receives	50	56	63	69	75	81	88	94	100	Associate:

Case 6

You receive	100	98	96	94	93	91	89	87	85	You:
Associate receives	50	54	59	63	68	72	76	81	85	Associate:

Next

Figure S14: Social value orientation slider measure at the end of the experiment.

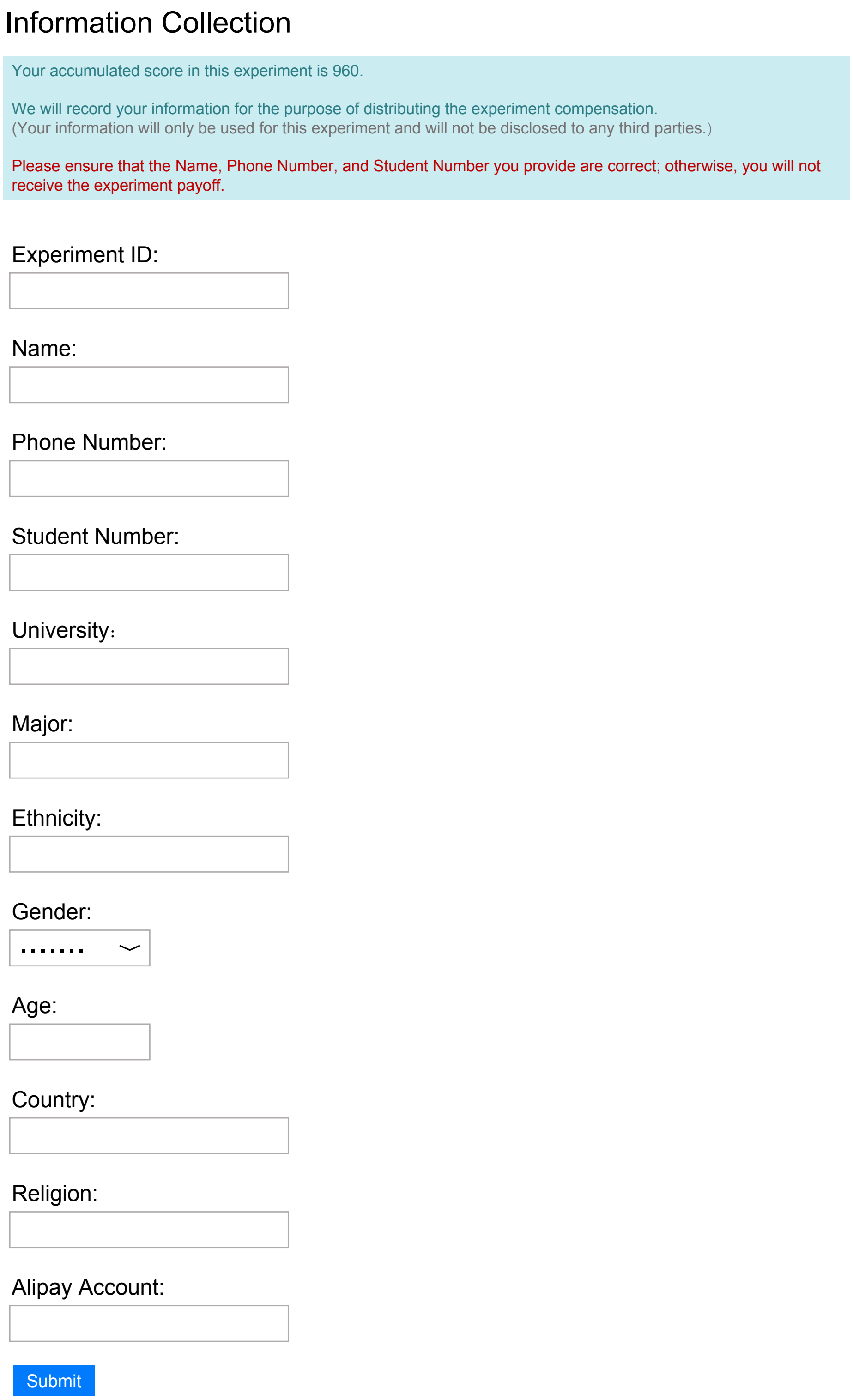

Information Collection

Your accumulated score in this experiment is 960.

We will record your information for the purpose of distributing the experiment compensation.
(Your information will only be used for this experiment and will not be disclosed to any third parties.)

Please ensure that the Name, Phone Number, and Student Number you provide are correct; otherwise, you will not receive the experiment payoff.

Experiment ID:

Name:

Phone Number:

Student Number:

University:

Major:

Ethnicity:

Gender:

Age:

Country:

Religion:

Alipay Account:

Submit

Figure S15: The information collection page at the end of the experiment.

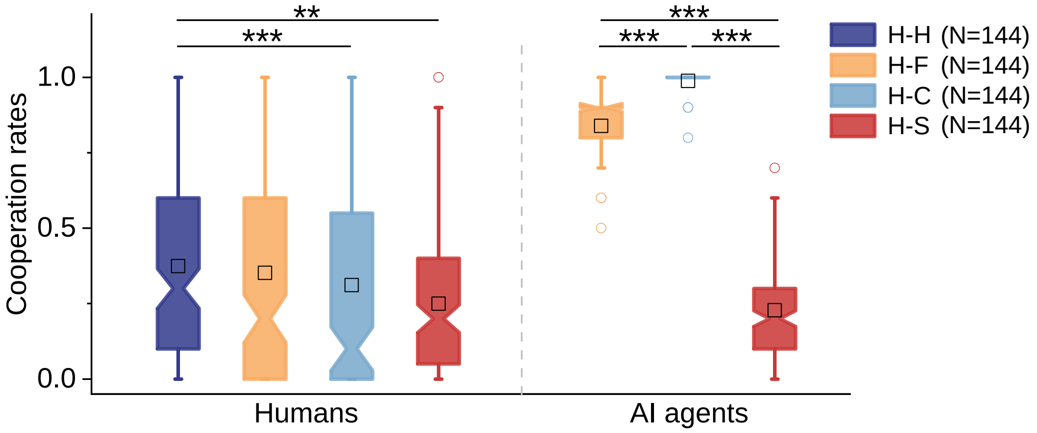

Figure S16: **Fair agents, unlike cooperative or selfish agents, are as effective as humans at eliciting human cooperation, thereby overcoming the machine penalty under the label-uninformed setting.** The left panel depicts participants' cooperation rates, while the right panel depicts the cooperation rates of agents. Participants' cooperation rates in the H-F treatment show no significant difference compared to those in the H-H treatment ($W = 11382$, $p = 0.1$, Cohen's $d = -0.07$). However, their cooperation rates in both the H-C and H-S treatments are significantly lower than those of the H-H treatment (H-C vs. H-H: $W = 12316$, $p < 0.01$, Cohen's $d = -0.19$; H-S vs. H-H: $W = 12912$, $p < 10^{-3}$, Cohen's $d = -0.46$). The cooperation rates of fair agents are significantly lower than those of cooperative agents ($W = 2898$, $p < 10^{-16}$, Cohen's $d = -1.53$), but significantly higher than those of selfish agents ($W = 20663$, $p < 10^{-16}$, Cohen's $d = 4.34$). Two-tailed Mann–Whitney U tests are used for pairwise comparisons. The robustness of these results is further corroborated by one-way ANOVA test and post-hoc analysis (Tables S10 and S11).

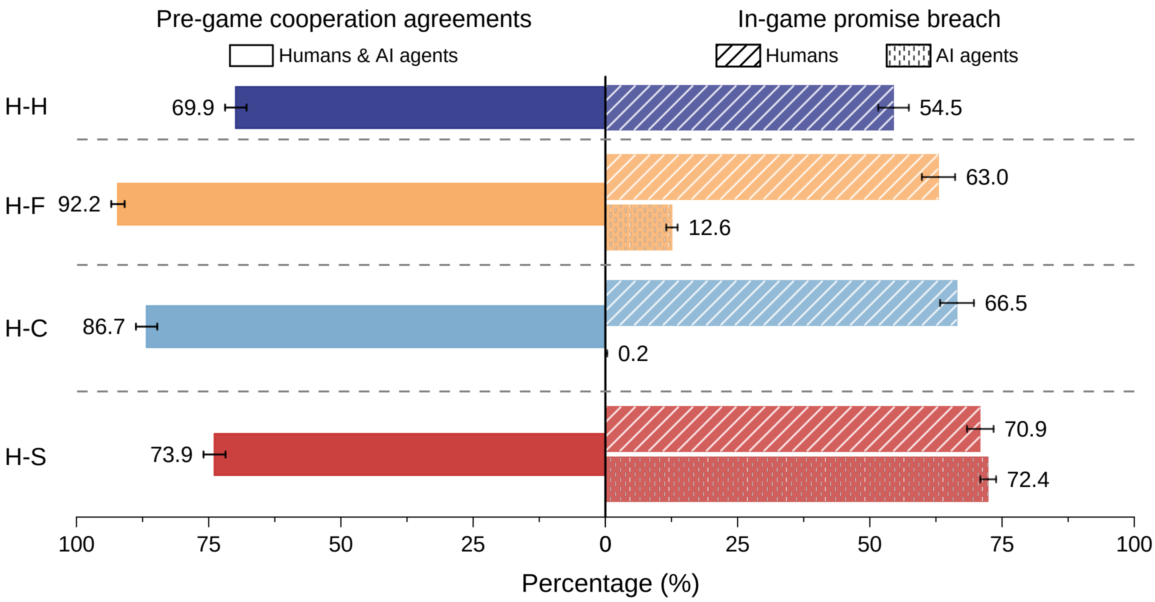

Figure S17: **All three types of agents frequently establish mutual cooperation agreements with humans during, the pre-game communication. However, humans often break cooperation promises, while fair agents also occasionally do so under the label-uninformed setting.** Participants are most likely to establish the cooperation agreements with fair agents at a significantly higher rate than participants in all the other treatments (H-F vs. H-H: $\chi^2 = 138.4$, $p < 10^{-15}$, Cohen's $h = 0.59$; H-F vs. H-C: $\chi^2 = 22.4$, $p < 10^{-5}$, Cohen's $h = 0.16$; H-F vs. H-S: $\chi^2 = 158.6$, $p < 10^{-15}$, Cohen's $h = 0.49$). However, during the games, participants typically break their promises, though they break promises significantly less frequently in the H-F treatment compared to the H-C and H-S treatments (H-F vs. H-C: $\chi^2 = 3.21$, $p = 0.07$, Cohen's $h = -0.07$; H-F vs. H-S: $\chi^2 = 16.05$, $p < 10^{-4}$, Cohen's $h = -0.17$). Fair agents break promises at a significantly higher rate than cooperative agents ($\chi^2 = 160.01$, $p < 10^{-15}$, Cohen's $h = 0.65$), but significantly lower than selfish agents ($\chi^2 = 883.12$, $p < 10^{-15}$, Cohen's $h = -1.31$). Two-sample proportions Z tests are used for pairwise comparisons. Statistical significance results of pairwise comparisons across each treatment and each dimension are provided in Tables S12.

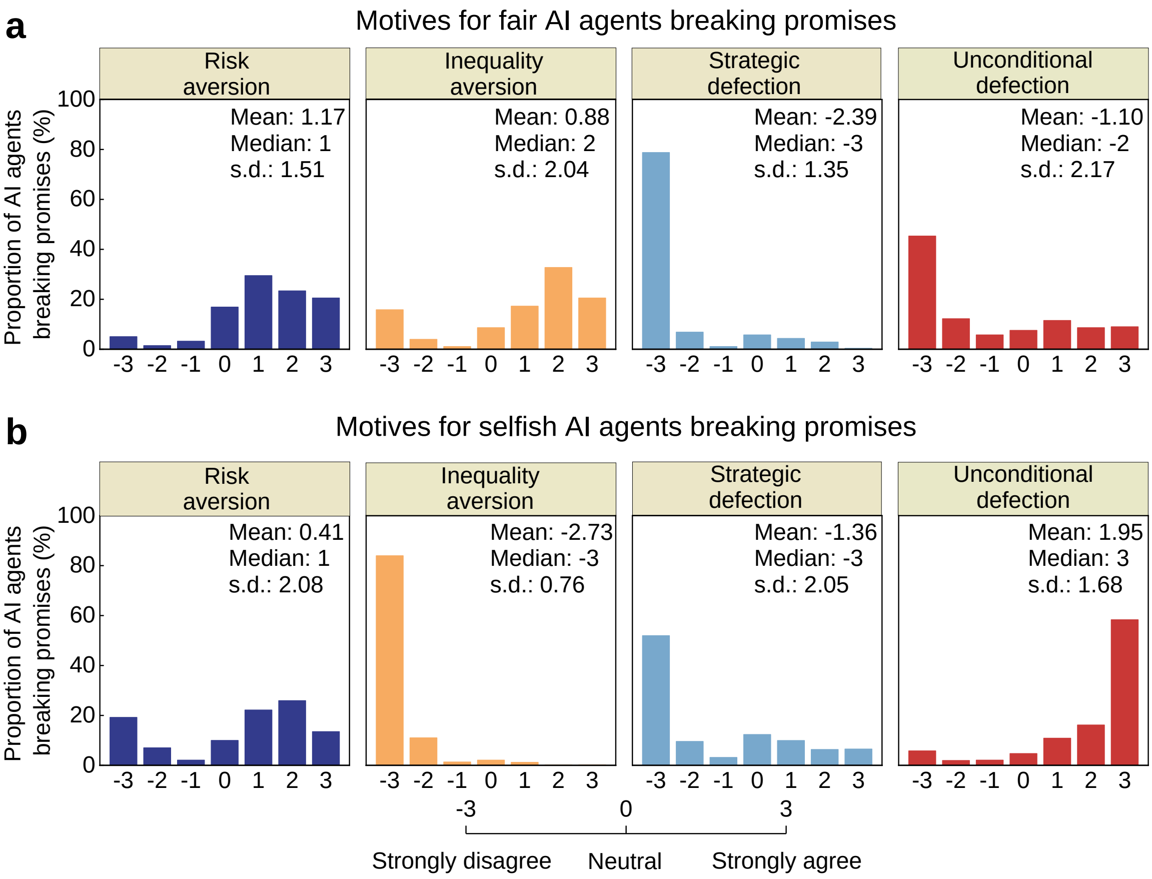

Figure S18: **Fair AI agents break promise due to risk aversion or inequality aversion, whereas selfish agents are driven mostly by unconditional defection and sometimes by risk aversion under the label-uninformed setting.** Panel A shows distributions of human experts' agreement levels for four potential motives for fair agents breaking promises, while Panel B shows those for selfish agents. For fair agents, promise-breaking is primarily motivated by risk aversion and inequality aversion, as the mean human agreement levels for these motives are significantly above zero (risk aversion: $V = 23134$, $p < 10^{-15}$; inequality aversion: $V = 22204$, $p < 10^{-6}$) while those for the other motives are significantly below zero (strategic defection: $V = 480.5$, $p < 10^{-15}$; unconditional defection: $V = 7194$, $p < 10^{-15}$). In contrast, selfish agents are driven mostly by unconditional defection ($V = 899109$, $p < 10^{-15}$) and sometimes by risk aversion ($V = 507689$, $p < 10^{-5}$), whereas human agreement levels for the other motives are significantly below zero (inequality aversion: $V = 3069.5$, $p < 10^{-15}$; strategic defection: $V = 127419$, $p < 10^{-15}$). The one-sample Wilcoxon signed-rank test is employed to determine whether the mean scores significantly differ from zero.

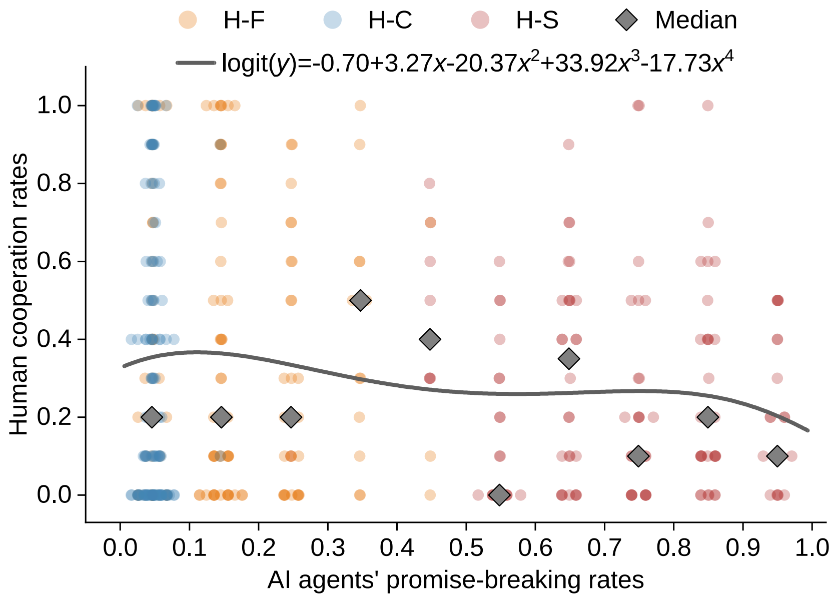

Figure S19: **Occasional treachery, exhibited by fair agents, is associated with the highest rates of human cooperation under the label-uninformed setting.** Scatter points depict the cooperation rates of individual participants when interacting with agents. The curve represents a generalized linear model (GLM) that incorporates data from all the interactions with three types of agents. This model treats human cooperation rates as the dependent variable, and includes linear (Estimate $\pm$ SE $= 3.27 \pm 1.37, z = 2.39, p = 0.02$), quadratic (Estimate $\pm$ SE $= -20.37 \pm 7.41, z = -2.75, p < 0.01$), cubic (Estimate $\pm$ SE $= 33.92 \pm 12.62, z = 2.69, p < 0.01$), and biguadratic (Estimate $\pm$ SE $= -17.73 \pm 6.63, z = -2.67, p < 0.01$) terms of agents promise-breaking frequency as independent variables. The curve shows an initial increase in human cooperation rates as the frequency of agents promise-breaking rises from zero, followed by a significant decrease at higher frequencies of agents promise-breaking.

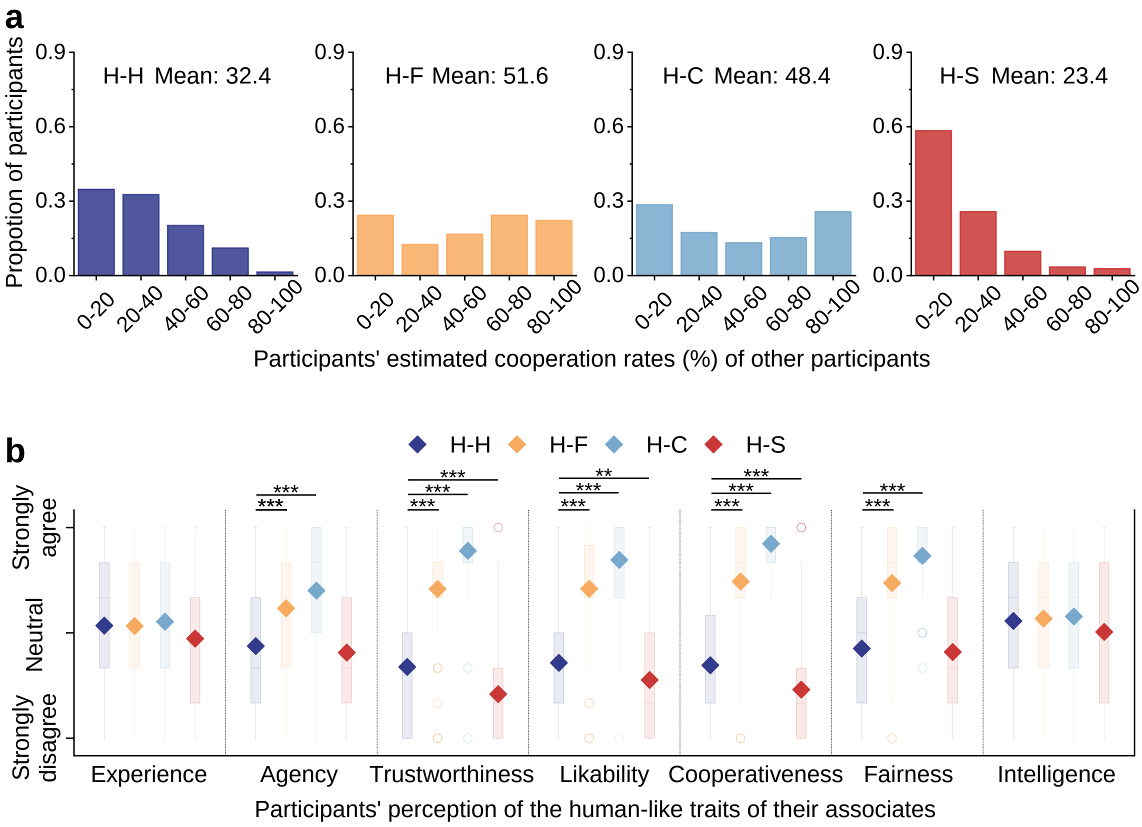

Figure S20: **Fair agents establish cooperative norms and are perceived as possessing experience, and intelligence, while also being viewed as more trustworthy, likable, cooperative, fair and agentic than humans under the label-uninformed setting.** The top panels depict participants' post-experiment estimations for cooperation from other participants in the same treatment, whereas the bottom panels depict participants' post-experiment agreement levels for various human-like traits of their associates in the treatment. Participants' estimations in both the H-F treatment and H-C are significantly higher than those in H-H and H-S treatments (H-F vs. H-H: $W = 6596$, $p < 10^{-7}$, Cohen's $d = 0.74$; H-F vs. H-S: $W = 15744$, $p < 10^{-14}$, Cohen's $d = 1.11$; H-C vs. H-H: $W = 7516$, $p < 10^{-4}$, Cohen's $d = 0.59$; H-C vs. H-S: $W = 14996$, $p < 10^{-11}$, Cohen's $d = 0.95$). Compared to humans, fair agents exhibit similar experience ($W = 10594$, $p = 0.75$, Cohen's $d = -0.01$) and intelligence ($W = 10144$, $p = 0.75$, Cohen's $d = 0.04$). In addition, they are seen as more trustworthy ($W = 3747$, $p < 10^{-20}$, Cohen's $d = 1.32$), likable ($W = 3932$, $p < 10^{-19}$, Cohen's $d = 1.27$), fair ($W = 4188.5$, $p < 10^{-18}$, Cohen's $d = 1.17$), cooperative ($W = 3447$, $p < 10^{-22}$, Cohen's $d = 1.41$), and agentic ($W = 6908.5$, $p < 10^{-6}$, Cohen's $d = 0.61$) than humans. Two-tailed Mann–Whitney U tests are used for pairwise comparisons. Statistical significance results of pairwise comparisons across each treatment and each dimension are provided in Tables S6.

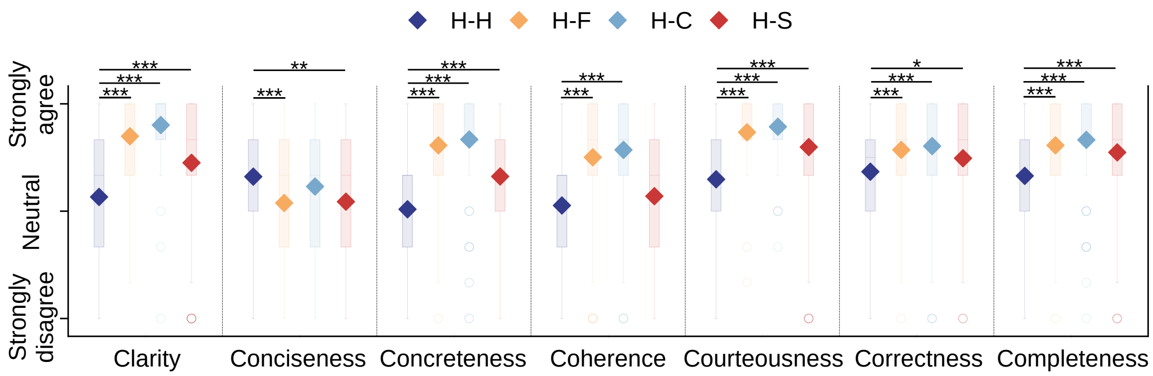

Figure S21: **Messages generated by fair agents are perceived as high quality and are viewed more positively in all aspects than those from humans under the label-uninformed setting.** Box plot depicts participants' post-experiment agreement levels for associates' communication quality according to the 7C standard, namely, clarity, conciseness, concreteness, coherence, courteousness, correctness, and completeness. Compared to humans, the messages generated by fair agents are perceived as having greater clarity ($W = 4774.5$, $p < 10^{-15}$, Cohen's $d = 1.13$), conciseness ($W = 12779.5$, $p < 10^{-3}$, Cohen's $d = 0.43$), concreteness ($W = 4290$, $p < 10^{-17}$, Cohen's $d = 1.17$), coherence ($W = 5617$, $p < 10^{-11}$, Cohen's $d = 0.84$), courteousness ($W = 5318$, $p < 10^{-13}$, Cohen's $d = 0.95$), correctness ($W = 7897.5$, $p < 10^{-3}$, Cohen's $d = 0.41$), and completeness ($W = 7206.5$, $p < 10^{-5}$, Cohen's $d = 0.61$) than those produced by humans. Two-tailed Mann–Whitney U tests are used for pairwise comparisons. Statistical significance results of pairwise comparisons across each treatment and each dimension are provided in Table S7.

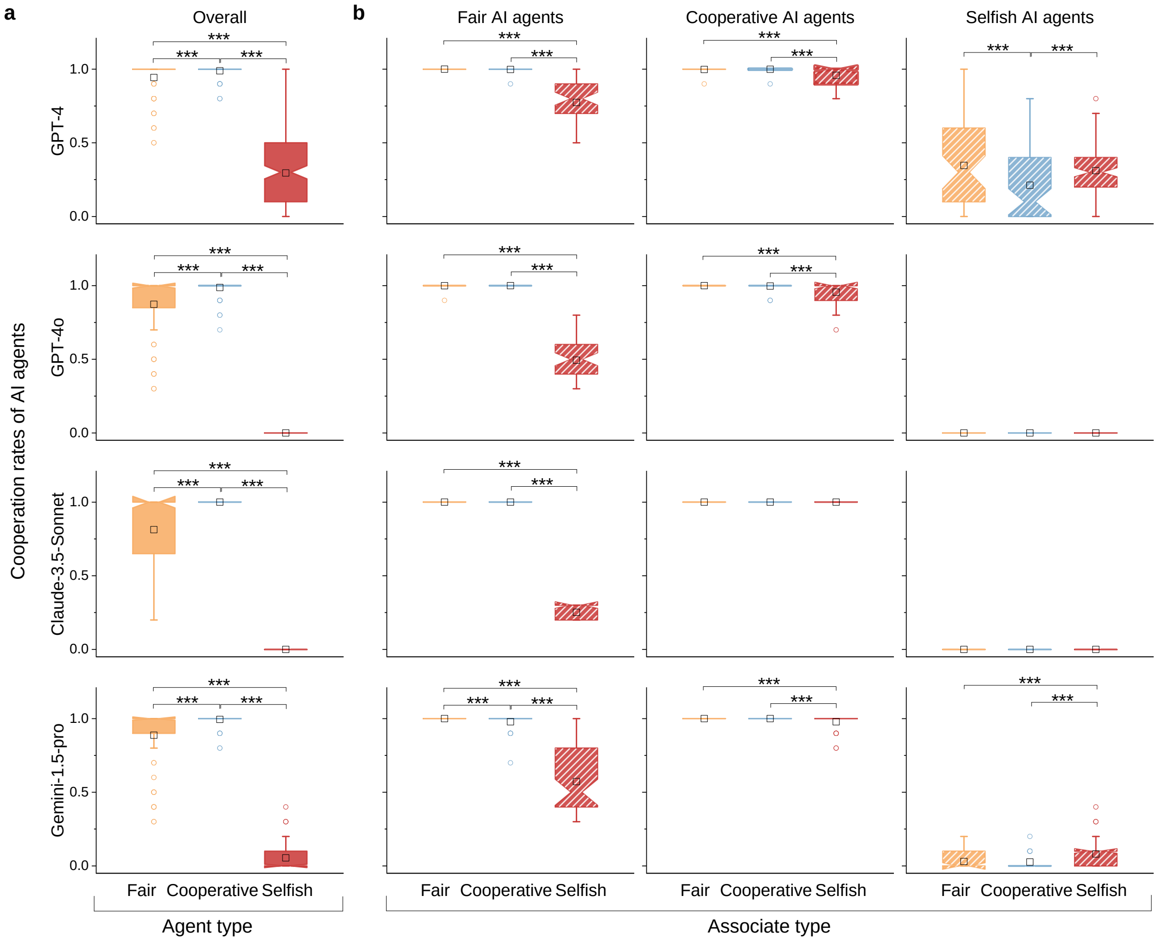

Figure S22: **Each LLM exhibits persona-aligned strategies, with cooperative agents consistently cooperating, selfish agents frequently defecting, and fair agents adapting decreasing cooperation against selfish agents.** Panel A shows the overall cooperation rates of agents across personas for four LLMs. Panel B breaks down the cooperation rate of fair, cooperative, and selfish agents when interacting with each associate type. Rows corresponding to LLMs (top to bottom: GPT-4, GPT-4o, Claude-3.5-Sonnet, and Gemini-1.5-pro). The cooperation rates of fair agents are significantly lower than those of cooperative agents, and significantly higher than those of selfish agents across all LLMs, with fair agents' cooperation rate against selfish agents significantly lower than against cooperative and fair agents. For fair agents interacting with selfish associates, GPT-4 shows the highest cooperation rates, while Claude-3.5-Sonnet shows the lowest.

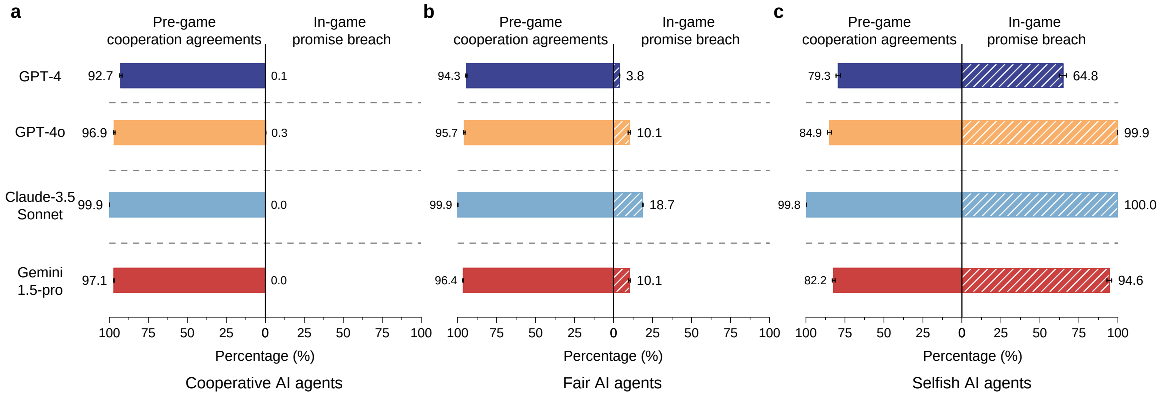

Figure S23: **Each LLM frequently establishes pre-game cooperation agreements across personas, with fair agents occasionally breaching in-game promises.** Panels A, B, and C show the percentages of pre-game cooperation agreements (left) and in-game promise breaches (right) for cooperative, fair, and selfish AI agents, respectively, across four LLMs: GPT-4, GPT-4o, Claude-3.5-Sonnet, and Gemini-1.5-Pro. Cooperative and fair agents consistently form cooperation agreements, with the cooperative agents showing minimal promise breaches and fair agents occasionally breaching, while selfish agents, despite forming promises, typically breach them. For fair agents, GPT-4 is the least likely to breach promises, while Claude-3.5-Sonnet breaches the most.

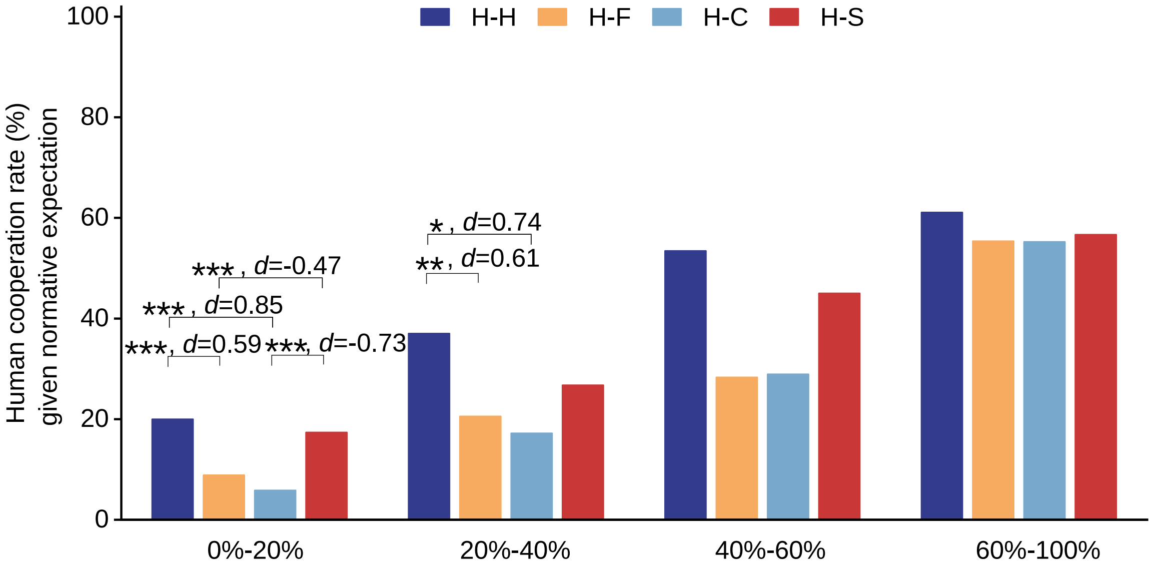

Figure S24: **Human normative expectations tend to be more effectively translated into decision-making when interacting with fellow humans than with AI agents under the label-uninformed setting.** Bars are grouped according to participants' normative expectations in each treatment, which are collected through post-experiment questionnaires. Within each group of normative expectations, participants' cooperation rates in H-H treatment are either significantly higher (for the normative expectation that falls within 0% – 20%: H-H vs. H-F: $z = 3.33$, $p < 10^{-3}$, Cohen's $d = 0.59$; H-H vs. H-C: $z = 3.59$, $p < 10^{-3}$, Cohen's $d = 0.85$; for 20% – 40%: H-H vs. H-F: $z = 2.66$, $p < 0.01$, Cohen's $d = 0.61$; H-H vs. H-C: $z = 2.46$, $p < 0.05$, Cohen's $d = 0.74$) or comparable to those in the H-C, H-F, and H-S treatments. However, except for normative expectations within the 0% – 20% interval, where human cooperation rates in the H-S treatment are significantly higher than those in the H-F and H-C treatments (H-S vs. H-F: $z = 3.48$, $p < 10^{-3}$, Cohen's $d = 0.47$; H-S vs. H-C: $z = 3.72$, $p < 10^{-3}$, Cohen's $d = 0.73$), there are no significant differences in human cooperation rates when interacting with different types of agents in other intervals. Due to the limited number of participants whose normative expectations fall within the 80% – 100% interval, the data of this interval are combined with those of the 60% – 80% interval. Two-tailed Mann–Whitney U tests are used for pairwise comparisons.

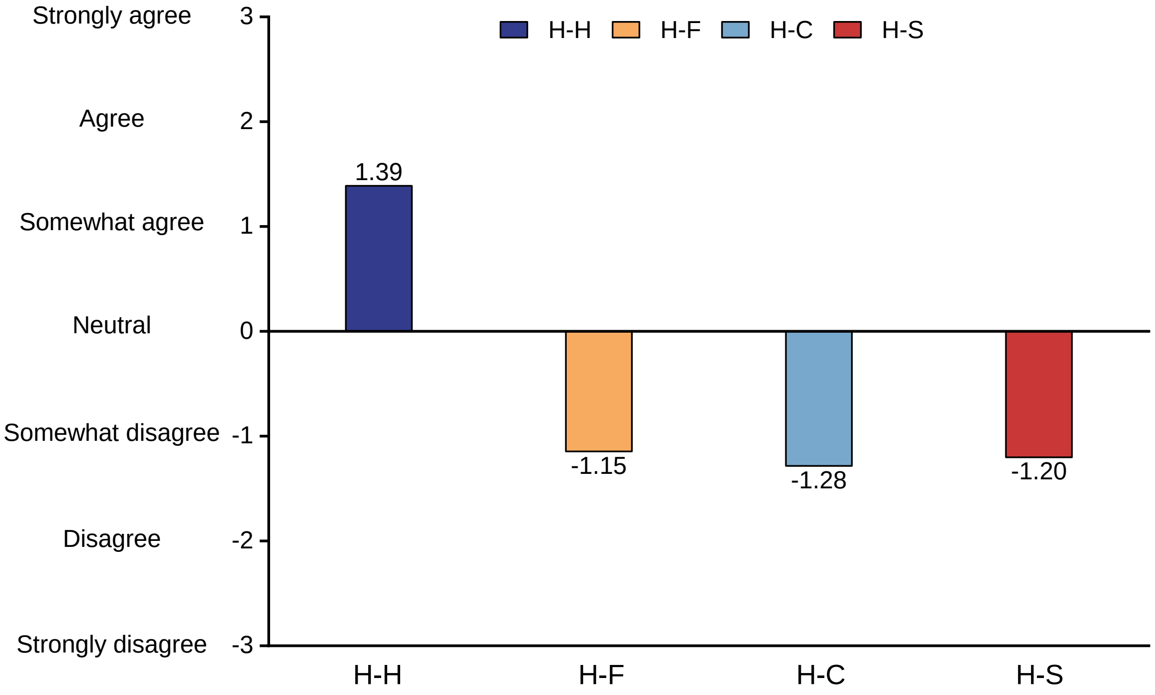

Figure S25: **Humans are able to differentiate between AI agents and humans, when they are aware of the potential involvement of artificial entities under the label-uninformed setting.** The panel depicts participants' agreement levels regarding whether their associates are humans or not, collected through a post-experiment questionnaire. In interactions with fellow humans, participants gave significantly higher than zero scores (H-H: $V = 7422$, $p < 10^{-13}$), indicating that they could accurately tell that their associates were human. In contrast, in interactions with AI agents, participants gave significantly lower than zero scores (H-F: $V = 1489.5$, $p < 10^{-10}$; H-C: $V = 1642.5$, $p < 10^{-10}$; H-S: $V = 1812$, $p < 10^{-9}$), indicating they could accurately tell that their associates were non-human. The one-sample Wilcoxon signed-rank test is employed to determine whether the mean scores significantly differ from zero.

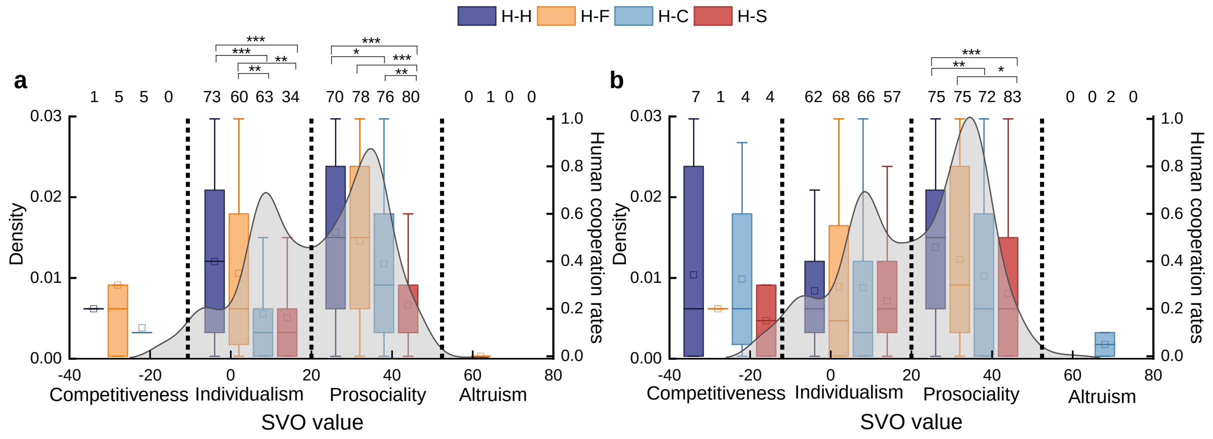

Figure S26: **Participants' cooperation frequency is modulated by their social value orientations under both label-informed and label-uninformed setting.** The cooperation frequency of participants, categorized according to their social value orientations (SVO), under label-informed (subplot A) and label-uninformed setting (subplot B). Most participants in our experiments are individualistic or prosocial. **Subplot A**: under the label-informed setting, individualistic participants exhibit significantly higher cooperation rates in the H-H and H-F treatments compared to the H-C and H-S treatments (H-H vs. H-C: $W = 3227$, $p < 10^{-4}$, Cohen's $d = 0.75$; H-H vs. H-S: $W = 3229$, $p < 10^{-4}$, Cohen's $d = 0.89$; H-F vs. H-C: $W = 2448.5$, $p < 10^{-2}$, Cohen's $d = 0.56$ H-F vs. H-S: $W = 2446$, $p < 10^{-2}$, Cohen's $d = 0.69$). Similarly, prosocial participants show the lowest cooperation rates in the H-S treatment, which is significantly lower than in the other three treatments (H-H vs. H-S: $W = 4315$, $p < 10^{-8}$, Cohen's $d = 1.10$; H-F vs. H-S: $W = 4603$, $p < 10^{-6}$, Cohen's $d = 0.95$; H-C vs. H-S: $W = 3934$, $p < 10^{-2}$, Cohen's $d = 0.61$). Their cooperation level in the H-C treatment shows some improvement—it is significantly lower than in H-H (H-H vs. H-C: $W = 3280$, $p < 0.05$, Cohen's $d = 0.39$), comparable to H-F. **Subplot B**: under the label-uninformed setting, individualistic participants show comparably low rates of cooperation, regardless of whether they interact with various types of agents or with other participants in the H-H treatment. On the contrary, prosocial participants show a significantly lower cooperation rates when they interact with cooperative agents (H-H vs. H-C: $W = 3387$, $p < 10^{-2}$, Cohen's $d = 0.37$) and selfish agents (H-H vs. H-S: $W = 4323.5$, $p < 10^{-4}$, Cohen's $d = 0.69$), as opposed to the humans vs humans scenario.

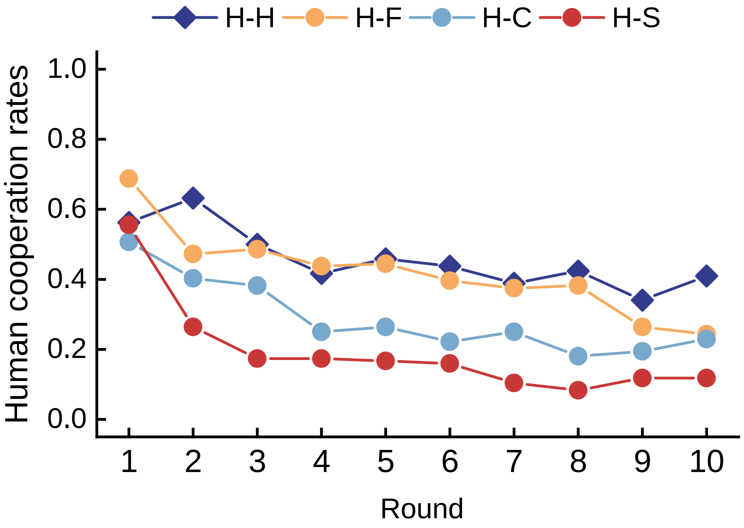

Figure S27: **The cooperation rate initially starts at a high level across all treatments but declines over time, with a slower decline and higher long-run cooperation when humans interact with other humans or fair agents.** This plot illustrates the evolution of cooperation frequency over 10 rounds in the label-informed setting. Although cooperation is initially high in all treatments, cooperation in the H-C and H-S treatments declines rapidly as the rounds progress, whereas cooperation in the H-H and H-F treatments declines more gradually and generally stabilizes at higher levels.

Table S1: Results of the one-way Analysis of Variance (ANOVA) assessing the impact of treatment types, i.e. human-human, human-cooperative AI agents, human-fair AI agents, and human-selfish AI agents, under the label-informed setting, on human cooperation rates. For detailed post-hoc analysis, please refer to Table S13.

Term	d.f.[a]	S.S[b]	MS[c]	F statistic	p-value
Condition	3	6.42	2.14	22.96	4.75e-14 ***
Residuals	572	53.27	0.09		

[a]Degrees of freedom
[b]Sum of squares
[c]Mean squares

Table S2: Pairwise comparisons of pre-game cooperation agreements and in-game promise breaches in four treatments under the label-informed setting: human-human (H-H), human-cooperative AI agents (H-C), human-fair AI agents (H-F), and human-selfish AI agents (H-S). The table examines the percentages of (1) pre-game cooperation agreements between humans and AI agents, (2) humans' in-game promise breaches, and (3) AI agents' in-game promise breaches, respectively. For each pairwise comparison, the table includes the percentages of the two treatments, the χ^2 statistic, and the p-value. Note that 'Percentage 1' and 'Percentage 2' refer to the results of the first and second treatments being compared, respectively.

Statistical test results are obtained through two-sample proportions Z test.

Dimension	Treatment	Percentage 1 (%)	Percentage 2 (%)	χ^2	p-value
Pre-game Cooperation Agreements	H-H vs. H-F	75.5(1086/1440)	87.5(1260/1440)	20.311	6.583e-6***
	H-H vs. H-C	75.5(1086/1440)	81.1(1167/1440)	0.196	0.657
	H-H vs. H-S	75.5(1086/1440)	79.7(1148/1440)	0.009	0.924
	H-F vs. H-C	87.5(1260/1440)	81.1(1167/1440)	22.697	1.896e-6***
	H-F vs. H-S	87.5(1260/1440)	79.7(1148/1440)	30.818	2.834e-8***
	H-C vs. H-S	81.1(1167/1440)	79.7(1148/1440)	0.565	0.452
Human In-Game Promise Breach	H-H vs. H-F	45.2(491/1086)	56.8(716/1260)	31.031	2.539e-8***
	H-H vs. H-C	45.2(491/1086)	68.2(796/1167)	120.530	<2.2e-16***
	H-H vs. H-S	45.2(491/1086)	79.2(909/1148)	273.810	<2.2e-16***
	H-F vs. H-C	56.8(716/1260)	68.2(796/1167)	32.945	9.482e-9***
	H-F vs. H-S	56.8(716/1260)	79.2(909/1148)	135.800	<2.2e-16***
	H-C vs. H-S	68.2(796/1167)	79.2(909/1148)	35.336	2.774e-9***
AI Agent In-Game Promise Breach	H-F vs. H-C	9.6(121/1260)	0.0(0/1167)	115.930	<2.2e-16***
	H-F vs. H-S	9.6(121/1260)	71.7(823/1148)	968.920	<2.2e-16***
	H-C vs. H-S	0.0(0/1167)	71.7(823/1148)	1295.0	<2.2e-16***

Table S3: Generalized linear models under the label-informed setting that take participants' cooperation rates as dependent variables, and various aspects of perceptions of AI agents, collected through post-experiment questionnaires, as independent variables. Separate models are constructed for the human-human treatment and the human-AI (H-C, H-S, and H-F) treatments, with the H-F treatment serving as the baseline. The generalized linear model indicates that prediction about other participants' cooperation is the most influential factor, regardless of whether participants interact with human or agent associates. However, the impact of other factors on human cooperation varies between human-human and human-AI treatments. In human-human treatment, the perceived trustworthiness and clarity of communication from fellow humans are significant predictors. In contrast, for the human-AI treatments, the perceived intelligence and fairness of AI agents, along with message conciseness are significant predictors.

	Dependent Variable: Human Cooperation Rates							
Model	Humans vs. Humans				Humans vs. AI agents			
	Coef.[a]	*S.E.*[b]	z value	pr(> $\|z\|$)	*Coef.*[a]	*S.E.*[b]	*z value*	pr(> $\|z\|$)
Intercept	-0.19	0.06	-3.32	9.17e-4 ***	-0.95	0.07	-13.64	<2e-16 ***
Clarity	0.24	0.08	3.19	1.42e-3 **	-0.03	0.05	-0.56	0.58
Conciseness	-0.03	0.07	-0.40	0.69	-0.10	0.04	-2.23	0.03 *
Concreteness	0.04	0.08	0.45	0.65	-0.02	0.06	-0.39	0.70
Coherence	-0.14	0.09	-1.59	0.11	-0.05	0.05	-0.93	0.35
Courteousness	-0.11	0.08	-1.38	0.17	-0.09	0.05	-1.82	0.07
Correctness	-0.04	0.08	-0.48	0.63	0.03	0.05	0.66	0.51
Completeness	-0.01	0.07	-0.19	0.85	-0.10	0.05	-1.75	0.08
Trustworthiness	0.37	0.11	3.19	1.43e-3 **	-0.04	0.11	-0.40	0.69
Intelligence	-0.13	0.07	-1.82	0.07	0.39	0.05	8.14	3.95e-16 ***
Cooperativeness	0.01	0.14	0.06	0.95	-0.12	0.12	-0.99	0.32
Likability	-0.22	0.13	-1.70	0.09	0.01	0.10	0.11	0.91
Fairness	0.03	0.09	0.34	0.73	0.23	0.08	3.00	2.74e-3 **
Agency	-0.05	0.08	-0.58	0.56	-0.08	0.05	-1.53	0.13
Experience	-0.01	0.07	-0.10	0.92	0.07	0.05	1.54	0.12
Prediction about other participants' cooperation	0.72	0.07	10.37	<2e-16 ***	0.85	0.04	19.03	<2e-16 ***
Treatment Effect H-C					0.04	0.10	0.43	0.67
Treatment Effect H-S					-0.31	0.13	-2.36	0.02 *
Null deviance			786.8				2284.1	
Residual deviance			565.8				1388.5	
AIC[c]			869.9				2120.4	
Obeservation			144				432	

[a]Coefficient

[b]Standard error

[c]Akaike information criterion

Table S4: Pairwise comparisons of participants' agreement levels for their associates' personality and mindfulness in four treatments under the label-informed setting: human-human (H-H), human-cooperative AI agents (H-C), human-fair AI agents (H-F), and human-selfish AI agents (H-S). The agreement levels are on 7-point Likert scales, ranging from -3 (strong disagreement) to 3 (strong agreement). For each pairwise comparison, the table includes the median and mean agreement levels of the two treatments, the W statistic, and the p-value. Note that the subscripts 1 and 2 after the median or mean indicate the first and second treatments, respectively. Statistical test results are obtained through two-tailed Mann-Whitney U test.

**Treatment**	**Dimension**	**Median1**	**Median2**	**Mean1**	**Mean2**	**W**	***p*-value**
H-H vs. H-F	Trustworthiness	-1	2	-0.590	1.451	4378.5	<2.2e-16***
	Intelligence	1	1	0.645	0.500	11465	0.114
	Cooperativeness	-1	2	-0.500	1.542	4145.5	<2.2e-16***
	Likability	-1	2	0.708	1.271	5047.5	2.384e-14***
	Fairness	0	2	-0.597	1.375	5721.5	2.23e-11***
	Agency	1	1	0.250	0.653	9173	0.086
	Experience	1	0	0.931	0.139	13373	1.612e-05***
H-H vs. H-C	Trustworthiness	-1	3	-0.590	2.424	1943.5	<2.2e-16***
	Intelligence	1	0	0.646	-0.104	12890	2.968e-4***
	Cooperativeness	-1	3	-0.500	2.659	1349.5	<2.2e-16***
	Likability	-1	2.5	0.708	2.083	2852.5	<2.2e-16***
	Fairness	0	2	-0.597	1.958	3919	<2.2e-16***
	Agency	1	2	0.250	1.347	6862	4.592e-07***
	Experience	1	0	0.931	-0.125	13597	3.762e-06***
H-H vs. H-S	Trustworthiness	-1	-2	-0.590	-1.424	12892	2.668e-4***
	Intelligence	1	1	0.646	0.611	10890	0.451
	Cooperativeness	-1	-2	-0.500	-1.361	12712	7.08e-4***
	Likability	-1	-1	0.708	-1.319	12859	3.352***
	Fairness	0	-1	-0.597	-1.014	13386	1.491e-05***
	Agency	1	0	0.250	-0.250	11980	0.021*
	Experience	1	0	0.931	0.083	13230	3.986e-05***
H-F vs. H-C	Trustworthiness	2	3	1.451	2.424	6142	1.597e-10***
	Intelligence	1	0	0.500	-0.104	12226	0.008**
	Cooperativeness	2	3	1.542	2.659	5367	6.417e-15***
	Likability	2	2.5	1.271	2.083	7109.5	1.533e-06***
	Fairness	2	2	1.375	1.958	7754.5	1.216***
	Agency	1	2	0.653	1.347	7713	1.283***
	Experience	0	0	0.139	-0.125	11254	0.205

Table S5: **Basic information on the conducted experimental sessions.** In total, 16 sessions were divided between eight treatments. Sessions were characterized by the order of experiment without communication (WoC) and with communication (WC), the number of interactions, attendance, the mean age of participants and its standard deviation, and the percentage of women. H-H, H-F, H-S, and H-C represent treatments conducted under the label-uninformed setting. H-HP, H-FP, H-SP, and H-CP represent treatments conducted under the label-informed setting.

Date	Treatment	Location	Order	Interactions	Participants	Mean age	SD age	%women
10 March 2024	H-H	Xi'an	WoC-WC	10-10	72	18.9	0.69	29.1
	H-H	Xi'an	WC-WoC	10-10	72	18.7	0.77	37.5
9 March 2024	H-F	Xi'an	WoC-WC	10-10	72	18.7	0.81	54.1
	H-F	Xi'an	WC-WoC	10-10	72	18.8	0.68	61.1
20 March 2024	H-C	Taiyuan	WoC-WC	10-10	72	19.9	1.06	61.1
	H-C	Taiyuan	WC-WoC	10-10	72	19.7	0.85	59.7
17 April 2024	H-S	Taiyuan	WoC-WC	10-10	72	19.1	1.04	34.7
	H-S	Taiyuan	WC-WoC	10-10	72	19.1	1.90	37.5
18 May 2024	H-HP	Kunming	WoC-WC	10-10	72	21.9	2.49	48.6
	H-HP	Kunming	WC-WoC	10-10	72	21.1	1.99	47.2
14,15 March 2024	H-FP	Xi'an	WoC-WC	10-10	72	25.1	1.92	77.7
	H-FP	Xi'an	WC-WoC	10-10	72	19.5	1.46	58.3
27 April 2024	H-CP	Kunming	WoC-WC	10-10	72	20.9	2.03	59.7
	H-CP	Kunming	WC-WoC	10-10	72	21.1	2.28	63.8
28 April 2024	H-SP	Kunming	WoC-WC	10-10	72	22.3	2.53	45.8
	H-SP	Kunming	WC-WoC	10-10	72	20.2	1.81	47.2

Table S6: Pairwise comparisons of participants' agreement levels for their associates' personality and mindfulness in four treatments under the label-uninformed setting: human-human (H-H), human-cooperative AI agents (H-C), human-fair AI agents (H-F), and human-selfish AI agents (H-S). The agreement levels are on 7-point Likert scales, ranging from -3 (strong disagreement) to 3 (strong agreement). For each pairwise comparison, the table includes the median and mean agreement levels of the two treatments, the W statistic, and the p-value. Note that the subscripts 1 and 2 after the median or mean indicate the first and second treatments, respectively. Statistical test results are obtained through two-tailed Mann-Whitney U test.

**Treatment**	**Dimension**	**Median1**	**Median2**	**Mean1**	**Mean2**	**W**	***p*-value**
H-H vs. H-F	Trustworthiness	-1	2	-0.972	1.250	3747	<2.2e-16***
	Intelligence	1	1	0.340	0.409	10144	0.748
	Cooperativeness	-1	2	-0.924	1.465	3447	<2.2e-16***
	Likability	-1	2	-0.854	1.257	3932	<2.2e-16***
	Fairness	0	2	-0.444	1.424	4188.5	<2.2e-16***
	Agency	-1	1	-0.375	0.701	6908.5	6.788e-07***
	Experience	1	0	0.208	0.194	10594	0.746
H-H vs. H-C	Trustworthiness	-1	3	-0.972	2.340	1469.5	<2.2e-16***
	Intelligence	1	1	0.340	0.465	9895	0.498
	Cooperativeness	-1	3	-0.924	2.542	932.5	<2.2e-16***
	Likability	-1	2	-0.854	2.076	1847	<2.2e-16***
	Fairness	0	2	-0.444	2.194	1948	<2.2e-16***
	Agency	-1	2	-0.375	1.201	5497	2.953e-12***
	Experience	1	0.5	0.208	0.319	9952	0.552
H-H vs. H-S	Trustworthiness	-1	-2	-0.972	-1.743	13029	1.027e-4***
	Intelligence	1	-2	0.340	0.028	11200	0.232
	Cooperativeness	-1	-2	-0.924	-1.618	12970	1.544e-04***
	Likability	-1	-2	-0.854	-1.340	12224	0.007**
	Fairness	0	-1	-0.444	-0.549	10884	0.459
	Agency	-1	-1	-0.375	-0.563	11002	0.363
	Experience	1	0	0.208	-0.167	11630	0.070
H-F vs. H-C	Trustworthiness	2	3	1.250	2.340	5538.5	7.116e-13***
	Intelligence	1	1	0.409	0.465	10130	0.733
	Cooperativeness	2	3	1.465	2.542	5988.5	2.926e-11***
	Likability	2	2	1.257	2.076	7302	6.308e-06***
	Fairness	2	2	1.424	2.194	7186	2.327e-06***
	Agency	1	2	0.701	1.201	8479	0.006**
	Experience	0	0.5	0.194	0.319	9912	0.514

Table S7: Pairwise comparisons of participants' agreement levels for their associates' communication quality in four treatments under the label-uninformed setting: human-human (H-H), human-cooperative AI agents (H-C), human-fair AI agents (H-F), and human-selfish AI agents (H-S). The agreement levels are on 7-point Likert scales, ranging from -3 (strong disagreement) to 3 (strong agreement). For each pairwise comparison, the table includes the median and mean agreement levels of the two treatments, the W statistic, and the p-value. Note that the subscripts 1 and 2 after the median or mean indicate the first and second treatments, respectively. Statistical test results are obtained through two-tailed Mann-Whitney U test.

**Treatment**	**Dimension**	**Median1**	**Median2**	**Mean1**	**Mean2**	**W**	***p*-value**
H-H vs. H-F	Clarity	1	2	0.396	2.097	4774.5	4.199e-16***
	Conciseness	1	1	0.965	0.229	12780	5.045e-04***
	Concreteness	1	2	0.056	1.840	4290	<2.2e-16***
	Coherence	1	2	0.159	1.507	5617	6.588e-12***
	Courteousness	1	2	0.889	2.208	5318	9.035e-14***
	Correctness	1.5	2	1.104	1.715	7897.5	3.067e-04***
	Completeness	1	2	0.986	1.840	7206.5	3.664e-06***
H-H vs. H-C	Clarity	1	3	0.396	2.409	3491.5	<2.2e-16***
	Conciseness	1	1	0.965	0.688	11136	0.266
	Concreteness	1	2	0.056	2.007	3570	<2.2e-16***
	Coherence	1	2	0.159	1.715	4836.5	1.343e-15***
	Courteousness	1	2.5	0.889	2.361	4564.5	<2.2e-16***
	Correctness	1.5	2	1.104	1.819	7321.5	8.254e-06***
	Completeness	1	2	0.986	1.993	6430	8.061e-09***
H-H vs. H-S	Clarity	1	2	0.396	1.354	7117.5	2.722e-06***
	Conciseness	1	1	0.965	0.264	12293	0.006**
	Concreteness	1	1	0.056	0.972	7125.5	2.867e-06***
	Coherence	1	1	0.159	0.417	9293.5	0.121
	Courteousness	1	2	0.889	1.792	6953.5	5.682e-07***
	Correctness	1.5	2	1.104	1.479	8819.5	0.024*
	Completeness	1	2	0.986	1.646	7885	2.910e-04***
H-F vs. H-C	Clarity	2	3	2.097	2.409	8388.5	0.002**
	Conciseness	1	1	0.229	0.688	8816.5	0.025*
	Concreteness	2	2	1.840	2.007	9229	0.089
	Coherence	2	2	1.507	1.715	9293.5	0.114
	Courteousness	2	2.5	2.208	2.361	9204.5	0.072
	Correctness	2	2	1.715	1.819	9739	0.351
	Completeness	2	2	1.840	1.993	9325.5	0.119

Table S8: Generalized linear models under the label-uninformed setting that take participants' cooperation rates as dependent variables, and various aspects of perceptions of AI agents, collected through post-experiment questionnaires, as independent variables. Separate models are constructed for the human-human treatment and the human-AI (H-C, H-S, and H-F) treatments, with the H-F treatment serving as the baseline. The generalized linear model indicates that prediction about other participants' cooperation is the most influential factor, regardless of whether participants interact with human or AI agent associates. However, the impact of other factors on human cooperation differs between human-human and human-AI treatments. In human-human treatment, the perceived likability, cooperativeness, as well as communication conciseness, clarity, courteousness, and completeness, are significant predictors. In contrast, for the human-AI treatments, the perceived intelligence, experience, cooperativeness, likability, agency of AI agents, along with communication completeness, concreteness, and correctness, are significant predictors.

	Dependent Variable: Human Cooperation Rates							
Model	Humans vs. Humans				Humans vs. AI agents			
	Coef.[a]	*S.E.*[b]	z value	pr(> $\|z\|$)	*Coef.*[a]	*S.E.*[b]	z *value*	pr(> $\|z\|$)
Intercept	-0.60	0.06	-10.01	<2e-16 ***	-1.09	0.07	-15.21	<2e-16 ***
Clarity	-0.49	0.09	-5.39	6.75e-8 ***	0.08	0.05	1.73	0.08
Conciseness	0.25	0.08	3.26	1.13e-3 **	0.04	0.04	1.02	0.31
Concreteness	0.18	0.09	1.79	0.07	-0.10	0.05	-1.99	0.04 *
Coherence	0.09	0.09	1.09	0.28	0.09	0.06	1.75	0.08
Courteousness	-0.26	0.08	-3.19	1.42e-3 **	-0.05	0.05	-0.97	0.33
Correctness	0.16	0.08	1.90	0.06	-0.18	0.06	-3.28	1.05e-03 **
Completeness	-0.27	0.08	-3.39	6.95e-4 ***	0.14	0.06	2.38	0.02 *
Trustworthiness	-0.07	0.11	-0.66	0.51	0.19	0.09	1.87	0.06
Intelligence	0.19	0.08	2.49	0.01 *	0.39	0.05	7.99	1.36e-15 ***
Cooperativeness	0.39	0.12	3.39	6.79e-4 ***	-0.39	0.11	-3.75	1.76e-4 ***
Likability	-0.24	0.12	-2.01	0.04 *	-0.24	0.09	-2.79	5.31e-3 **
Fairness	-0.19	0.09	-1.95	0.05	0.05	0.07	0.68	0.49
Agency	-0.11	0.08	-1.37	0.17	-0.11	0.06	-2.04	0.04 *
Experience	0.08	0.08	1.01	0.31	0.15	0.05	3.01	2.57e-3 **
Prediction about other participants' cooperation	0.66	0.07	9.24	<2e-16 ***	1.06	0.05	21.93	<2e-16 ***
Treatment Effect H-C					-0.02	0.09	-0.17	0.87
Treatment Effect H-S					0.11	0.13	0.82	0.41
Null deviance			650.9				2569.9	
Residual deviance			434.2				1679.0	
AIC[c]			751.5				2348.4	
Obeservation			144				432	

[a]Coefficient

[b]Standard error

[c]Akaike information criterion

Table S9: Pairwise comparisons of participants' agreement levels for their associates' communication quality in four treatments under the label-informed setting: human-human (H-H), human-cooperative AI agents (H-C), human-fair AI agents (H-F), and human-selfish AI agents (H-S). The agreement levels are on 7-point Likert scales, ranging from -3 (strong disagreement) to 3 (strong agreement). For each pairwise comparison, the table includes the median and mean agreement levels of the two treatments, the W statistic, and the p-value. Note that the subscripts 1 and 2 after the median or mean indicate the first and second treatments, respectively. Statistical test results are obtained through two-tailed Mann-Whitney U test.

**Treatment**	**Dimension**	**Median1**	**Median2**	**Mean1**	**Mean2**	**W**	**p-value**
H-H vs. H-F	Clarity	2	2	1.319	1.764	8738	0.017*
	Conciseness	2	1	1.354	1.159	11468	0.106
	Concreteness	1	2	0.951	1.868	7328.5	8.04e-06***
	Coherence	2	2	1.111	1.451	9462.5	0.185
	Courteousness	2	2	1.326	2.000	7939	3.175e-4***
	Correctness	2	2	1.639	1.493	11125	0.265
	Completeness	2	2	1.465	1.799	9214.5	0.086
H-H vs. H-C	Clarity	2	3	1.319	2.486	5574.5	6.443e-13***
	Conciseness	2	2	1.354	1.618	9256	0.102
	Concreteness	1	2	0.951	2.201	5681.5	4.594e-12***
	Coherence	2	2	1.111	1.750	7917.5	3.324e-4***
	Courteousness	2	3	1.326	2.535	5050	1.297e-15***
	Correctness	2	2	1.639	1.972	8636	0.009**
	Completeness	2	2	1.465	2.201	6941	2.845e-07***
H-H vs. H-S	Clarity	2	2	1.319	1.083	11013	0.349
	Conciseness	2	1	1.354	0.986	11862	0.029*
	Concreteness	1	2	0.951	1.236	9645	0.292
	Coherence	2	1	1.111	0.750	11924	0.024*
	Courteousness	2	2	1.326	1.472	10202	0.809
	Correctness	2	2	1.639	1.500	11251	0.191
	Completeness	2	2	1.465	1.556	10491	0.855
H-F vs. H-C	Clarity	2	3	1.764	2.486	7076.5	4.955e-07***
	Conciseness	1	2	1.159	1.618	8157	0.001**
	Concreteness	2	2	1.868	2.201	8324.5	0.002**
	Coherence	2	2	1.451	1.750	8614.5	0.009**
	Courteousness	2	3	2.000	2.535	6914	9.869e-08***
	Correctness	2	2	1.493	1.972	7900.5	2.557e-04***
	Completeness	2	2	1.799	2.201	7905.5	1.948e-04***

Table S10: Results of the one-way Analysis of Variance (ANOVA) assessing the impact of treatment types i.e. human-human, human-cooperative AI agents, human-fair AI agents, and human-selfish AI agents, under the label-uninformed setting, on human cooperation rates. For detailed post-hoc analysis, please refer to SI, Table S11.

Term	d.f.[a]	S.S[b]	MS[c]	F statistic	p-value
Condition	3	1.30	0.4341	4.277	0.005 **
Residuals	572	58.06	0.1015		

[a]Degrees of freedom
[b]Sum of squares
[c]Mean squares

Table S11: Summary of Tukey Honest Significant Difference (HSD) post-hoc analysis for the impact of treatment types i.e. human-human (H-H), human-cooperative AI agents (H-C), human-fair AI agents (H-F), and human-selfish AI agents (H-S), under the label-uninformed setting, on human cooperation rates. The table shows for each pairwise comparison, the difference in the mean human cooperation rate, the lower confidence bound (LCB), the upper confidence bound (UCB), and the p-value. There is no significant difference between H-F and H-H treatments, H-F and H-C treatments, H-H and H-C treatments, H-S and H-C treatments. However, there are significant differences between H-S and H-F treatments, as well as H-S and H-H treatments.

Comparison			Difference	LCB[a]	UCB[b]	p-value
H-F	vs.	H-C	0.041	-0.056	0.138	0.695
H-H	vs.	H-C	0.063	-0.034	0.159	0.334
H-S	vs.	H-C	-0.062	-0.159	0.035	0.354
H-H	vs.	H-F	0.022	-0.075	0.119	0.935
H-S	vs.	H-F	-0.103	-0.199	-0.006	0.032 *
H-S	vs.	H-H	-0.125	-0.222	-0.029	0.005 **

[a]Lower confidence bound

[b]Upper confidence bound

Table S12: Pairwise comparisons of pre-game cooperation agreements and in-game promise breaches in four treatments under the label-uninformed setting: human-human (H-H), human-cooperative AI agents (H-C), human-fair AI agents (H-F), and human-selfish AI agents (H-S). The table examines the percentages of (1) pre-game cooperation agreements between humans and AI agents, (2) humans' in-game promise breaches, and (3) AI agents' in-game promise breaches, respectively. For each pairwise comparison, the table includes the percentages of the two treatments, the χ^2 statistic, and the p-value. Note that the subscripts 1 and 2 after the percentage indicate the first and second treatments, respectively. Statistical test results are obtained through two-sample proportions Z test.

Dimension	Treatment	Percentage1 (%)	Percentage2 (%)	χ^2	p-value
Pre-game Cooperation Agreements	H-H vs. H-F	69.9(1006/1440)	92.2(1327/1440)	138.380	<2.2e-16***
	H-H vs. H-C	69.9(1006/1440)	86.7(1249/1440)	57.101	4.139e-14***
	H-H vs. H-S	69.9(1006/1440)	73.9(1064/1440)	0.024	0.876
	H-F vs. H-C	92.2(1327/1440)	86.7(1249/1440)	22.440	2.168e-6***
	H-F vs. H-S	92.2(1327/1440)	73.9(1064/1440)	158.560	<2.2e-16***
	H-C vs. H-S	86.7(1249/1440)	73.9(1064/1440)	74.348	<2.2e-16***
Human In-Game Promise Breach	H-H vs. H-F	54.5(548/1006)	63.0(836/1327)	16.886	3.969e-5***
	H-H vs. H-C	54.5(548/1006)	66.5(830/1249)	33.147	8.546e-9***
	H-H vs. H-S	54.5(548/1006)	70.9(754/1064)	58.835	1.715e-14***
	H-F vs. H-C	63.0(836/1327)	66.5(830/1249)	3.210	0.073
	H-F vs. H-S	63.0(836/1327)	70.9(754/1064)	16.047	6.178e-5***
	H-C vs. H-S	66.5(830/1249)	70.9(754/1064)	4.978	0.026*
AI Agent In-Game Promise Breach	H-F vs. H-C	12.6(167/1327)	0.2(2/1249)	160.010	<2.2e-16***
	H-F vs. H-S	12.6(167/1327)	72.4(770/1064)	883.120	<2.2e-16***
	H-C vs. H-S	0.2(2/1249)	72.4(770/1064)	1344.0	<2.2e-16***

Table S13: Summary of Tukey Honest Significant Difference (HSD) post-hoc analysis for the impact of treatment types, i.e. human-human (H-H), human-cooperative AI agents (H-C), human-fair AI agents (H-F), and human-selfish AI agents (H-S), under the label-informed setting, on human cooperation rates. The table shows for each pairwise comparison, the difference in the mean human cooperation rate, the lower confidence bound (LCB), the upper confidence bound (UCB), and the p-value. There is no significant difference between H-F and H-H treatments. However, there are significant differences between H-F and H-C treatments, H-H and H-C treatments, H-S and H-C treatments, H-S and H-F treatments, as well as H-S and H-H treatments.

Comparison			Difference	LCB[a]	UCB[b]	p-value
H-F	vs.	H-C	0.131	0.038	0.223	0.002 **
H-H	vs.	H-C	0.169	0.076	0.261	2.010e-05 ***
H-S	vs.	H-C	-0.097	-0.189	-0.004	0.037*
H-H	vs.	H-F	0.038	-0.054	0.131	0.713
H-S	vs.	H-F	-0.227	-0.319	-0.134	<0.001 ***
H-S	vs.	H-H	-0.265	-0.358	-0.173	<0.001 ***

[a]Lower confidence bound

[b]Upper confidence bound

References

[1] Duffy J and Feltovich N. Do actions speak louder than words? an experimental comparison of observation and cheap talk. *Games Econ Behav* 2002; **39**: 1–27.

[2] Murphy RO, Ackermann KA, Handgraaf MJ. Measuring social value orientation. *Judgm Decis Mak* 2011; **6**: 771–81.

[3] Deutsch M. The effect of motivational orientation upon trust and suspicion. *Hum Relat* 1960; **13**: 123–39.
